# OMIDIENT: Multiomics Integration for Cancer by Dirichlet Auto-Encoder Networks

**DOI:** 10.1038/s42003-026-10578-1

**Published:** 2026-07-06

**Authors:** Negar Safinianaini, Niko Välimäki, Roman Bresson, Alexandra Gorbonos, Kristiina Rajamäki, Lauri A. Aaltonen, Pekka Marttinen

**Affiliations:** 1 Dept. of Computer Science, Aalto University, Helsinki, Finland; 2 Dept. of Medical and Clinical Genetics, University of Helsinki, Helsinki, Finland; 3 Applied Tumor Genomics Research Program, Research Programs Unit, University of Helsinki, Helsinki, Finland; 4 Mohamed bin Zayed University of Artificial Intelligence, Abu Dhabi, UAE; 5 iCAN Digital Precision Cancer Medicine Flagship, University of Helsinki, Helsinki, Finland

**Keywords:** Machine learning, Computational models

## Abstract

To achieve a more comprehensive understanding of cancer, novel computational methods are required for the integrative analysis of data from different molecular layers, such as genomics, transcriptomics, and epigenomics. Here, we present an innovative multi-omics integrative method that performs unsupervised representation learning, referred to as OMIDIENT: multiOMics Integration for cancer by DIrichlet auto-ENcoder neTworks. OMIDIENT provides a natural framework for modeling sparse and compositional latent representations by employing a deep generative model, where the latent space is distributed as the product of Dirichlet distributions. Applied to five different cancers, we demonstrate that OMIDIENT outperforms the top state-of-the-art unsupervised multi-omics integrative analysis approaches in clustering, classification, and reconstruction of missing data using mRNA expression data, DNA methylation data, and microRNA expression data. Furthermore, we provide interpretability analyses for OMIDIENT that not only support its improved performance, but also offer valuable insights into the underlying structure captured by the learned representations.

## Abstract (web summary)

OMIDIENT, a multiomics deep generative model based on the product of Dirichlet, leverages sparse and compositional representations; for 4 cancers, it outperforms state-of-the-art methods in clustering, classification, and missing data reconstruction.

## Introduction

Cancer is a complex disease in which various biological alterations give rise to characteristic patterns; discovering those structures is bound to have clinical relevance and can subsequently improve cancer treatment. Due to the heterogeneous nature of cancer, it is vital to identify specific patterns and clinical treatment options for different patients. Therefore, it is of utmost importance to provide a space where one can efficiently analyze the multimodal high-dimensional data. Integrating multiomics facilitates a comprehensive understanding of cellular diversity by combining data from different molecular layers, such as genomics, transcriptomics, and epigenomics. Each omics layer captures a different aspect of cellular function, and integration allows us to model the complex, interconnected nature of biology more effectively than any single omics type alone.

Multi-omics integration has been extensively studied in cancer research^1–5^, with various methods proposed. In MOGONET^6^, a graph-based supervised learning is proposed, while in MOCSS^7^, an unsupervised—learning without access to the subtyping labels—approach is adopted. Classical approaches have also been explored, such as NEMO^8^, which employs a neighborhood-based clustering strategy, and DeFusion^9^, which formulates multiomics integration as a non-negative matrix factorization problem. Each of these methods addresses a specific downstream task, such as cancer subtyping or survival analysis. However, given the complexity of multiomics data, the typically limited number of tumor samples, and the exploratory nature of subtyping—which often involves discovering previously unknown subtypes—we are particularly motivated to adopt an unsupervised learning approach. Building on the growing field of *representation learning*^10^, our goal is to learn deep, hierarchically abstract features that capture shared and modality-specific biological patterns, where more abstract concepts are higher in the hierarchy and deeper in the neural network. By embedding biological complexity into structured latent spaces, these compact and informative representations improve performance in downstream tasks such as subtyping and the prediction of survival and drug response.

To address these challenges, we propose a multi-omic integration method for unsupervised representation learning, referred to as OMIDIENT: MultiOMics Integration for cancer by DIrichlet auto-ENcoder neTworks. OMIDIENT, to the best of our knowledge, is a novel probabilistic model for learning latent representations of tumor samples. It is inspired by two state-of-the-art methods for representation learning from different fields: MOCSS, a method for multi-omic data integration^7^, which captures omics-specific and omics-shared representations, and Dirichlet variational autoencoder^11^, which naturally finds distinct groups among the samples. We generalize the Dirichlet variational autoencoder by modeling the latent representations as a *product* of Dirichlet distributions, enabling the model to organize samples along multiple dimensions through distinct subsets of the latent space. This provides a natural framework for learning sparse and compositional structure in the data. Our method improves cancer subtyping and reconstruction of missing data. Dividing features into distinct groups allows the model to capture structured biological or functional relationships, such as variability in gene sets, pathways, or phenotypes. In this way, the internal variation remains interpretable and biologically meaningful, which we validate by analyzing the interpretability of our method.

Recent methods have shown strong performance in unsupervised learning tasks. We evaluated OMIDIENT on five public cancer datasets: breast, colon, colorectal, liver, and kidney cancer, learning representations using mRNA expression, DNA methylation, and microRNA expression data. Across these datasets, OMIDIENT outperformed existing approaches in cancer subtyping, evaluated through clustering and classification based on the learned representations, and achieved superior performance in missing omic data reconstruction. OMIDIENT is designed to flexibly integrate additional modalities if available.

## Results

### Framework of OMIDIENT

**Fig. 1:**
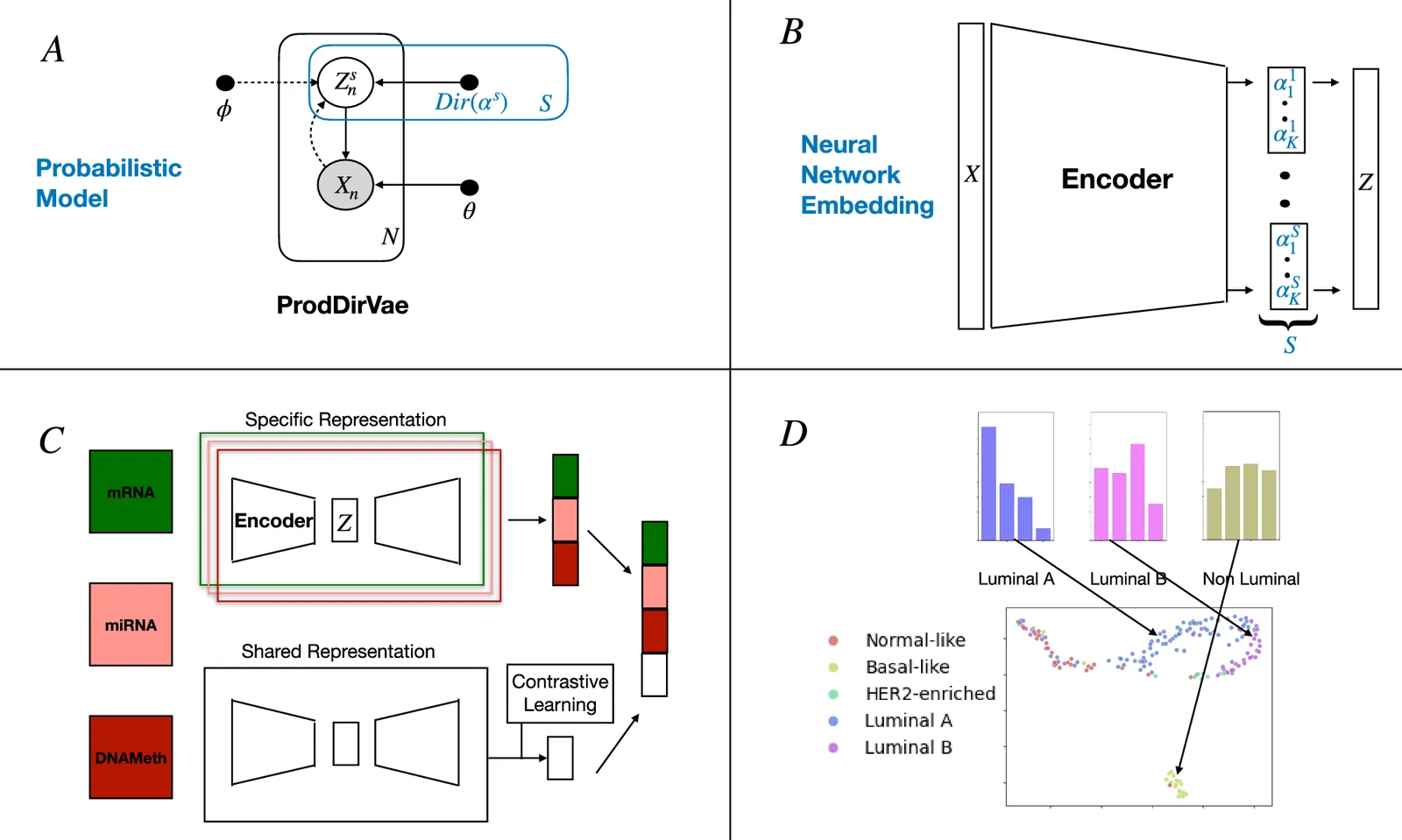
OMIDIENT’s framework overview. **A** Our innovative probabilistic graphical model, which models the latent representation *Z* as belonging to *S* distinct groups. **B** The corresponding unfolded computational graph of the neural network architecture. **C** Overview of learning omic-specific and shared representations. **D** Demonstration of the utility of the learned representation *Z*, where we perform BRCA subtyping based on *Z*. For clarity, we only show group *S* = 1 for mRNA omics, which clearly separates Luminal A and B subtypes, while the basal-like subtype exhibits a near-uniform distribution, reflecting its non-luminal characteristics.

We introduce OMIDIENT, shown in Fig. 1, an unsupervised multi-omics integration framework for tumor representation learning—these representations can be used for downstream tasks in cancer analysis, such as subtyping. Specifically, we introduce an innovative probabilistic model, referred to as ProdDirVae and illustrated in part A in Fig. 1, to capture *S* distinct groups of tumor characteristics. Each omic dataset is modeled by ProdDirVae to capture omic-specific representations. For shared omic representations, we use contrastive learning similarly to MOCSS^7^, since contrastive learning is a state-of-the-art, powerful machine learning tool for capturing shared representations of multiple modalities^12^. We conduct comprehensive ablation studies to demonstrate the necessity of different components in OMIDIENT. A major advantage of ProdDirVae is its robustness toward the high-dimensionality of multiomics, given limited tumor samples.

Motivated by the task of cancer subtyping, we apply clustering and classification using the learned tumor representations. In addition, we reconstruct each omic modality from these representations to assess their generative quality. For subtyping, we evaluate model performance using ground-truth labels across five public datasets: (1) breast invasive carcinoma (BRCA), (2) colon adenocarcinoma (COAD), (3) colorectal cancer (CRC), (4) liver hepatocellular carcinoma (LIHC), and (5) kidney renal clear cell carcinoma (KIRC). To demonstrate the effectiveness and robustness of OMIDIENT, we conduct extensive experiments across multiple clustering, classification, and missing data reconstruction tasks. Our analysis incorporates three omic types—mRNA expression, DNA methylation, and miRNA expression—capturing complementary and comprehensive views of tumor biology.

For the details of our method and the datasets, see Section “Methods.”

### Clustering and classification

For the downstream task of cancer subtyping, we compared the clustering performance of OMIDIENT with top unsupervised multi-omics integration frameworks, i.e., MOFA2^13^, a factor analysis tool for multi-omics integration, MOCSS^7^, a deep learning and contrastive learning framework, NEMO^8^, a neighborhood-based multi-omics clustering, DeFusion^9^, a non-negative matrix factorization method, Concerto^14^, a contrastive learning tool, MultiVI^15^, and a joint omic probabilistic model. Moreover, from machine learning literature, we adapted other probabilistic models similar to OMIDIENT to assess how variations in model design affect performance; namely, LassoAE^16^, VAE^17^, a variational autoencoder-based multi-omics integration, LapDirVae^18^, a Laplace approximated Dirichlet autoencoder-based multi-omics integration, and GamDirVae^11^, a Gamma approximated Dirichlet autoencoder-based multi-omics integration.

We evaluated all the methods on five datasets and six clustering and classification scenarios. BRCA data with five subtypes, COAD data with four subtypes, LIHC data with cancer and normal samples, KIRC with two subtypes, and CRC with four or two classes. Microsatellite instability (MSI) arises through defective DNA mismatch repair in the tumors, causing mutations particularly at repetitive DNA sequences called “microsatellites”; the MSI classification system is relevant for CRC prognosis and treatment^19^. Consequently, for the CRC with four classes, we used the following groups: (1) microsatellite instability (MSI), (2) microsatellite stability (MSS), (3) ambiguous tumors, and (4) normal samples. For the CRC with two classes, we used the following groups within consensus molecular subtype (CMS): (1) CMS1 group containing microsatellite instability immune, 14%, hypermutated, microsatellite unstable, and strong immune activation and (2) other CMS groups and normal samples. For the details of the datasets and classes, see Section “Methods.”

We evaluated K-means clustering using normalized mutual information (NMI) based on the ground-truth class labels for each dataset. As shown in Table 1, our method, OMIDIENT, outperforms all methods on average. We also applied the k-nearest neighbors (k-NN) algorithm, reporting classification accuracy in the latent space for methods that provided explicit representations^11^. The k-NN results, shown in Tables 2 and 3, confirm again that OMIDIENT, on average, outperforms other methods. For the data with high class imbalance that is CRC with two clusters, we also performed AUC-ROC and permutation test on the k-NN results, see Supplementary Section 3.

**Table 1:** K-means performance, measured by NMI, for C classes based on the learned representations

Method \ Data	BRCA	COAD	LIHC	KIRC	CRC	CRC	Average %
	C = 5	C = 4	C = 2	C = 2	C = 4	C = 2	
MOCSS (AE)	46%	37%	17%	24%	26%	5%	26%
MOFA2	30%	17%	14%	1%	30%	**10%**	17%
LassoAE	44%	38%	20%	20%	34%	3%	26%
Concerto	41%	19%	12%	20%	17%	**10%**	20%
MultiVI	46%	14%	6%	10%	26%	2%	17%
NEMO	39%	3%	1%	–	–	–	14%
DeFusion	37%	18%	**32%**	1%	17%	1%	17%
VAE	39%	**44%**	11%	2%	**38%**	5%	23%
LapDirVae	41%	34%	6%	11%	33%	5%	21%
GamDirVae	42%	33%	12%	10%	21%	5%	20%
OMIDIENT (ours)	**50%**	36%	21%	**54%**	33%	**10%**	**34%**

In all the upcoming tables, we highlight results with bold values. Bold values are the outperforming ones.

**Table 2:** k-NN performance, measured by accuracy, for C classes based on the learned representations

Method/data	BRCA	COAD	LIHC	KIRC	CRC	CRC	Average %
	C = 5	C = 4	C = 2	C = 2	C = 4	C = 2	
MOCSS (AE)	70%	62%	89%	**100%**	75%	69%	77%
MOFA2	71%	**68%**	91%	86%	77%	**88%**	80%
LassoAE	70%	62%	95%	**100%**	62%	75%	77%
Concerto	68%	54%	89%	54%	62%	69%	66%
MultiVI	**82%**	31%	89%	**100%**	69%	**88%**	76%
NEMO	–	–	–	–	–	–	–
DeFusion	70%	65%	**97%**	78%	58%	65%	72%
VAE	70%	62%	89%	93%	69%	69%	75%
LapDirVae	70%	62%	89%	93%	**81%**	83%	80%
GamDirVae	73%	46%	89%	87%	**81%**	69%	76%
OMIDIENT (ours)	77%	62%	95%	**100%**	**81%**	75%	**82%**

In all the upcoming tables, we highlight results with bold values. Bold values are the outperforming ones.

**Table 3:** k-NN performance, measured by F1-score, for C classes based on the learned representations

Method/data	BRCA	COAD	LIHC	KIRC	CRC	CRC	Average %
	C = 5	C = 4	C = 2	C = 2	C = 4	C = 2	
MOCSS (AE)	48%	38%	72%	**100%**	46%	54%	59%
MOFA2	63%	23%	48%	92%	30%	**69%**	54%
LassoAE	49%	38%	82%	**100%**	42%	49%	60%
Concerto	40%	17%	67%	20%	19%	41%	34%
MultiVI	**75%**	16%	80%	95%	37%	71%	62%
NEMO	–	–	–	–	–	–	–
DeFusion	41%	34%	**86%**	50%	54%	46%	52%
VAE	48%	**39%**	72%	48%	45%	54%	51%
LapDirVae	51%	37%	72%	48%	55%	64%	54%
GamDirVae	50%	28%	72%	46%	**59%**	54%	51%
OMIDIENT (ours)	67%	37%	82%	81%	55%	59%	**64%**

In all the upcoming tables, we highlight results with bold values. Bold values are the outperforming ones.

Overall, the results indicate that OMIDIENT produces highly informative latent representations for both unsupervised and supervised downstream tasks. As shown in Table 1, OMIDIENT achieves the highest average NMI score and performs particularly well on the BRCA, KIRC, and CRC datasets. On average, its clustering performance exceeds that of competing methods by approximately 8 percentage points. Similarly, Table 2 shows that OMIDIENT achieves the highest average classification accuracy with the k-NN classifier, while maintaining strong performance across all datasets. This trend is further corroborated by the F1-scores reported in Table 3, where OMIDIENT consistently outperforms the competing approaches.

Regarding NEMO, note that the tool does not provide any learned representations; hence, the k-NN classification task was not applicable. Moreover, NEMO could not be run on KIRC data due to its high dimensionality in the gene expression data. Finally, NEMO could not be executed on the CRC dataset due to an error in the original software, which we did not attempt to resolve within the scope of this study.

### Reconstruction of missing data

We evaluated OMIDIENT on the task of missing data reconstruction, comparing it to MOCSS^7^, the top-performing state-of-the-art method as identified in Tables 1 and 2. Note that when an omic or omic feature is missing, the framework can be used as is, with no changes to the framework.

To simulate missing data scenarios, we conducted experiments shown in Tables 4–7, where in one set, we completely omitted an omic data from the test set; in the other set, we randomly removed features across all three omics types, ranging from 10% to 50% missingness. We used Pearson and Spearman correlations to evaluate the closeness of the reconstructed omics to the original omics.

**Table 4:** Reconstruction of missing data evaluated by Pearson (P) & Spearman (S) correlations for BRCA

Method/missingness	10%		30%		50%		miRNA		mRNA		DNAmethyl	
	P	S	P	S	P	S	P	S	P	S	P	S
MOCSS (AE)	93%	92%	95%	92%	94%	92%	89%	77%	61%	57%	60%	55%
OMIDIENT (ours)	**99%**	**99%**	**99%**	**99%**	**99%**	**99%**	**99%**	**99%**	**99%**	**99%**	**99%**	**99%**

In all the upcoming tables, we highlight results with bold values. Bold values are the outperforming ones.

**Table 5:** Reconstruction of missing data evaluated by Pearson (P) & Spearman (S) correlations for COAD

Method/missingness	10%		30%		50%		miRNA		mRNA		DNAmethyl	
	P	S	P	S	P	S	P	S	P	S	P	S
MOCSS (AE)	81%	79%	85%	80%	83%	78%	98%	95%	64%	61%	65%	61%
OMIDIENT (ours)	**100%**	**99%**	**99%**	**99%**	**100%**	**98%**	**99%**	**99%**	**99%**	**98%**	**100%**	**99%**

In all the upcoming tables, we highlight results with bold values. Bold values are the outperforming ones.

**Table 6:** Reconstruction of missing data evaluated by Pearson (P) & Spearman (S) correlations for LIHC

Method/missingness	10%		30%		50%		miRNA		mRNA		DNAmethyl	
	P	S	P	S	P	S	P	S	P	S	P	S
MOCSS (AE)	88%	85%	92%	88%	89%	84%	97%	91%	32%	31%	72%	68%
OMIDIENT (ours)	**99%**	**99%**	**99%**	**99%**	**99%**	**99%**	**98%**	**95%**	**99%**	**98%**	**100%**	**99%**

In all the upcoming tables, we highlight results with bold values. Bold values are the outperforming ones.

**Table 7:** Reconstruction of missing data evaluated by Pearson (P) & Spearman (S) correlations for KIRC

Method/missingness	10%		30%		50%		miRNA		mRNA		DNAmethyl	
	P	S	P	S	P	S	P	S	P	S	P	S
MOCSS (AE)	99%	**98%**	99%	**98%**	**99%**	**98%**	96%	**95%**	96%	**96%**	95%	95%
OMIDIENT (ours)	**100%**	**98%**	**100%**	**98%**	**99%**	**98%**	**98%**	**95%**	**98%**	95%	**100%**	**97%**

In all the upcoming tables, we highlight results with bold values. Bold values are the outperforming ones.

We evaluated our method and MOCSS on four datasets: BRCA data, COAD data, LIHC data, and KIRC. Tables 4–7 show that our method consistently outperforms MOCSS in all datasets and missing data scenarios, except for some values in the KIRC data, which are mostly on par with MOCSS. We also measured the *p*-value for all the computed correlations; for MOCSS, the *p*-values ranged from 8.74e − 252 to 3.26e − 08, and for OMIDIENT (our method), the *p*-values ranged from 1.30e − 269 to 2.14e − 155.

### Interpretability

#### Biological interpretation of learned representations for colorectal cancer

In this section, we consider biological interpretations of the learned representations for the CRC data, since for those data, we have access to multiple annotations.

First, we interpret the learned representations in terms of the MSI and MSS grouping that are established subtypes in colorectal cancer^20–25^. We compare the UMAP illustration of the samples’ learned representations by the top models in Tables 1 and 2. The samples include both tumors and normal colon samples, i.e., entirely nonneoplastic tissue. Although our method (OMIDIENT) and LapDirVae show similar performance on k-means and k-NN metrics for the CRC data, the UMAP visualization reveals that tumor samples form denser and more distinctly separated clusters with our approach. While additional genomic or clinical annotations are needed to fully interpret these groups, the results suggest that our method may uncover novel subgroups, particularly within the MSS subtype. For the details of the UMAP comparison, see Supplementary Subsection 2.1.

Next, we interpret the learned representations by CMS groupings, a gene expression-based CRC classification system associated with biological mechanisms of tumorigenesis and with patient prognosis^26^. Namely, we compare CMS1 versus other CMS subtypes^26^ because it most closely corresponds to the MSI subtype, i.e., most MSI tumors tend to be CMS1. It is important to note that MSI subtypes are more robustly defined in CRC, whereas CMS2-4 are inherently less well-characterized and exhibit substantial overlap along a biological continuum. This instability explains why CMS2-4 are not well classified across different methods; see more details in Supplementary Subsection 2.2.

Applying UMAP on top of the learned representations, our method, as reported in Table 2, achieves a more distinct and well-separated grouping of CMS1 samples. In contrast, MOCSS places the CMS1 samples in close proximity to, or overlapping with, other CMS groups, indicating less discriminative representations. Finally, although MultiVI and LapDirVae achieve high accuracy in Table 2, the UMAP plots reveal poor clustering of CMS1, suggesting that the seemingly high accuracy may be an artifact caused by class imbalance. This is further supported by its low NMI score. For UMAP visualizations, see Supplementary Subsection 2.2.

Moreover, we annotate the samples by gender and tumor stages. Our method distinguishes the genders clearly. Note that, due to the use of UMAP for dimensionality reduction, not all learned dimensions are visualized. As a result, some female samples appear close to non-tumor tissue. We also observe that the “normal-adjacent” (male) tumor cluster in OMIDIENT has fewer MSI tumors, and the “normal-distant” (female) tumor cluster has more MSI tumors. This observation is consistent with the well-established finding that MSI is more prevalent in colorectal cancers (CRCs) among females than males. It also appears OMIDIENT can distinguish the MSI subgroup among women quite well into its own half of the female cluster, while in the male cluster, MSI tumors are fully scattered around. Finally, the adenoma samples cluster close to stages I and II, which is biologically plausible; corresponding UMAP visualizations are provided in Supplementary Subsection 2.3. To mitigate batch effects, we first applied removeBatchEffect from the limma package in R and subsequently reran our method on the corrected data. The resulting clustering and classification performance remained consistent with the previously reported results, as further illustrated in Supplementary Subsection 2.3.

**Fig. 2:**
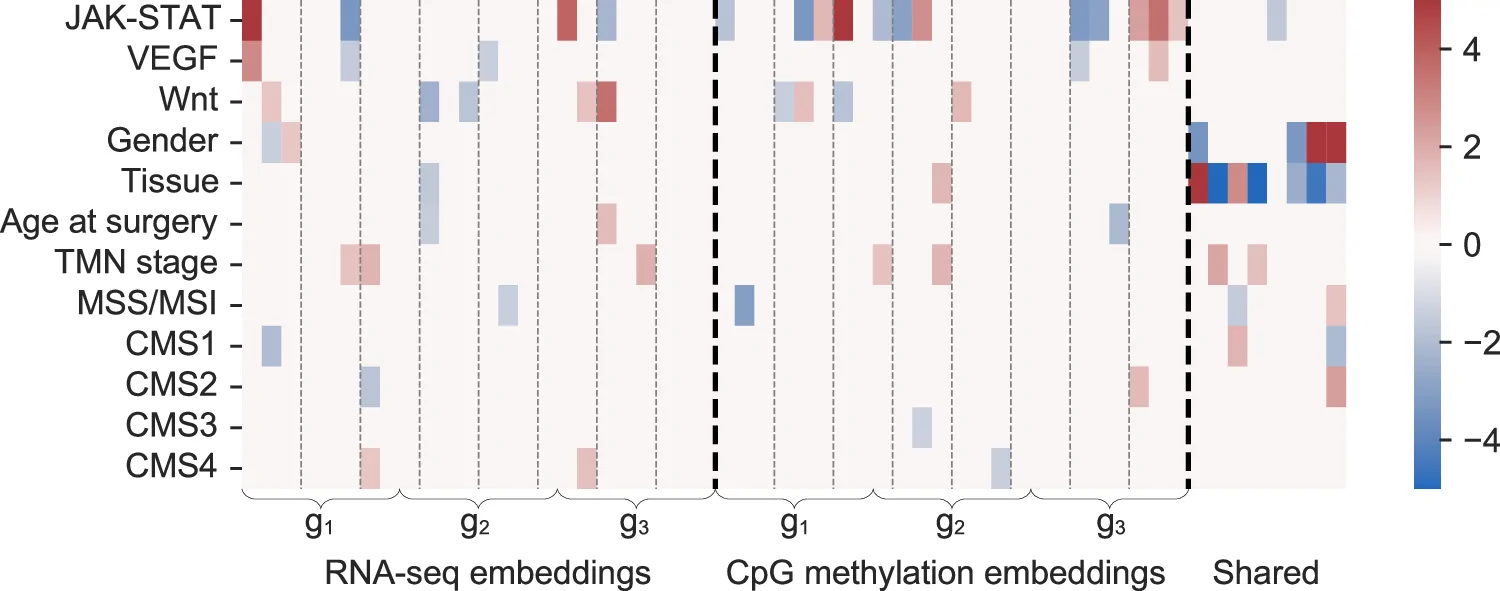
OMIDIENT-derived latent representations reveal associations with cancer-relevant pathways, features, and subtypes in colorectal cancer (CRC). The heatmap, based on *n* = 62, shows the sign(coefficient) *log10(*P*) values from each association test, where the coefficient gives the positive/negative direction. The columns represent the 56 latent representation dimensions, and the rows correspond to different annotations. Each omic is divided into 3 groups (g1, g2, g3), revealing the grouping characteristic of the OMIDIENT framework, which leverages the product of Dirichlet distributions. The JAK–STAT, VEGF, and Wnt represent gene set enrichment analysis of KEGG signaling pathways. The gender, tissue, age at surgery, TMN stage, and MSS/MSI are clinical background information. The CMS1–4 labels were predicted from the RNA-seq data. Red color denotes positive correlation, and black color denotes negative correlation.

In addition to the annotations above, we perform pathway enrichment analysis. Figure 2 shows the associations between latent representation values and different annotations. The shared representation is associated with gender, tissue, and TMN stage. The omic-specific representations make the most significant contributions to gene set enrichment analysis of key signaling pathways. The representations for mRNA and DNA methylation appear to be complementary and partially represent orthogonal signals from the two different omics. There were no associations with age at surgery. Some signaling pathways are known to be involved in different molecular subtypes of CRC^26^, with JAK-STAT, VEGF, and Wnt given here as a representative example. The gene set mRNA enrichment analysis showing CRC canonical pathways in tumor samples is inferred by using clusterProfiler (v4.10.0; org.Hs.eg.db v3.18.0), KEGG (accessed on May 15, 2025), and genes ranked by their gene expression correlation with each latent representation.

#### Biomarker and stage analysis for breast cancer

We first examine tumor stages in Breast cancer. Despite the heterogeneity of this cancer, we still found some patterns regarding the later stages of the disease; for more details, see Supplementary Subsection 2.4.

Next, we aim to analyze how slight changes in each feature affect the distances between neighboring tumors in the latent space. The result of this analysis shows how sensitive a feature is to infinitesimal movements of neighboring tumors. For this purpose, we apply a gradient-based analysis of the k-NN algorithm. The process for a given test sample *x*_*test*_ is the following. We obtain the nearest neighbors *x*_1_ to *x*_*k*_. Let *d*_*i*_ = ∣∣*z*_test_ − *z*_*i*_∣∣ be the euclidean distance between the latent representation of the test sample and that of its ith nearest neighbor—the choice of euclidean distance is due to that being used in the k-NN). For each neighbor, and for each input feature (i.e., taken before the encoder) *ϕ*_*j*_, we compute $\frac{\partial {d}_{i}}{\partial {\phi }_{j}}$, the gradient of the distance with regards to that feature. This gives us a mapping where each feature is associated with the influence it has on the distance. If this gradient is large in absolute value, the feature is highly influential.

**Fig. 3:**
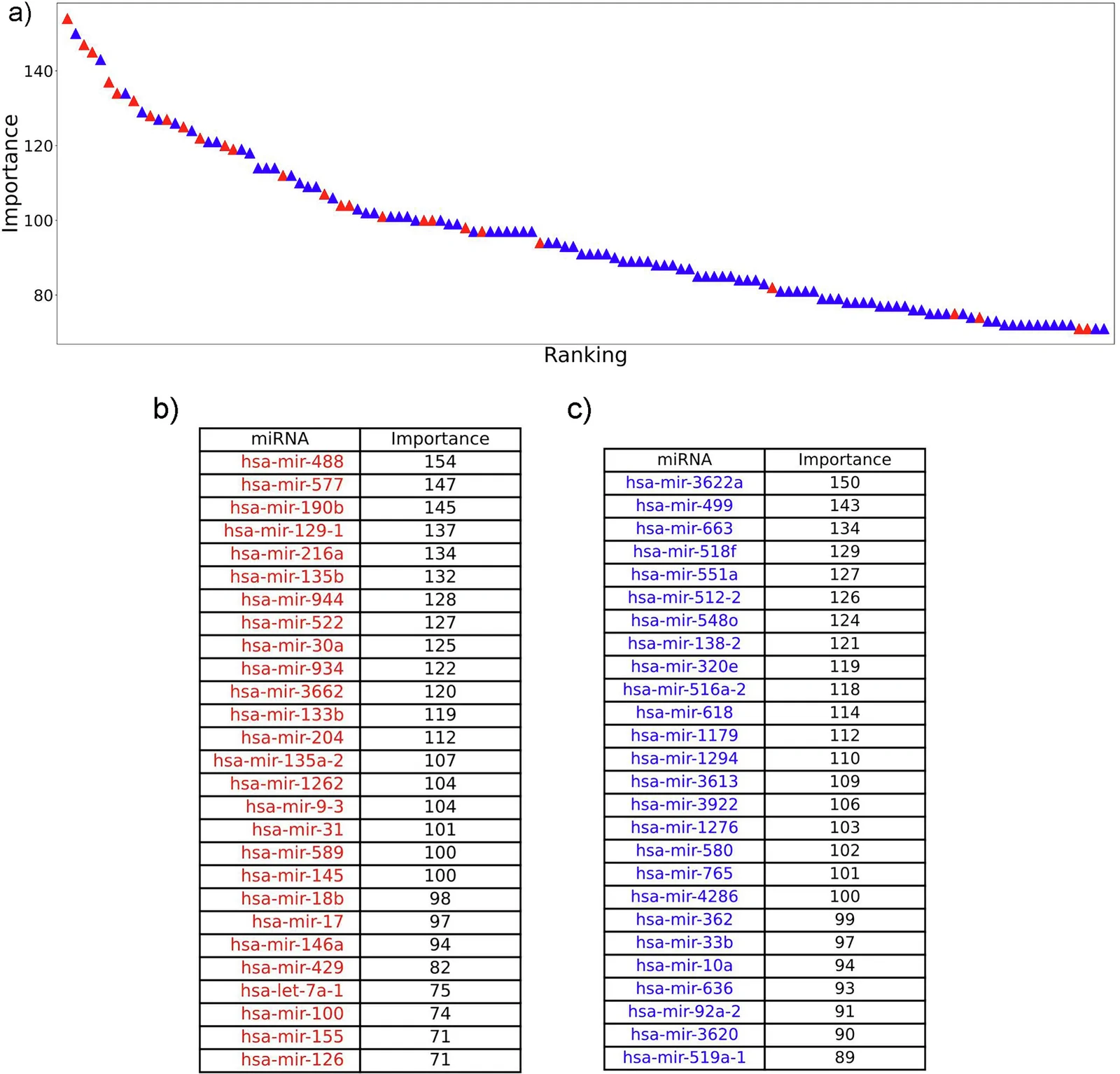
OMIDIENT rediscovers known miRNA biomarkers for breast cancer (BRCA) and identifies novel candidates associated with neoplasms and related conditions. The figure in panel (**a**) shows 126 biomarkers detected by OMIDIENT, ranked by importance, with red indicating previously reported biomarkers and blue indicating the top 26 novel (to the best of our knowledge) ones. The red and blue miRNAs are listed in the corresponding tables in panels (**b**) and (**c**).

After computing the gradients, we performed the following steps to report the top BRCA biomarkers. (1) For each tumor-neighbor pair, we identify the top 5% of features with the highest gradients (amounting to 125 features out of the total 2503). Since we consider 5 neighbors for each of the 44 tumor samples, this results in a total of 44 × 5 × 125 = 27,500 top-gradient features. (2) For each feature, we count how often it appears among the list of top-gradient features (each feature can appear at most 44 × 5 times, once per neighbor-example pair). We then select the 125 features (5%) with the highest overall frequencies across these sets. Except for one feature, the top features are all from miRNA omics. Figure 3 shows these top influential features, which are miRNA features. Despite the analysis being about the contribution of features to neighboring and similarities of tumor samples, we can observe that many of them are established BRCA biomarkers. Here, we review the miRNAs that OMIDIENT detected as influential, in which the state-of-the-art methods have also reported as biomarkers, as shown in red in Fig. 3. Note that most of the top-hits are on previously reported associations, which further validates our method. For a figure with all biomarkers in one plot, see Supplementary Subsection 2.5.

Starting from miRNAs with higher importance (ranks starting from one), as seen in panel b) in Fig. 3, hsa-mir-488 is involved in regulating the metastasis of breast cancers^27^. Tumor size, tumor stage, and lymphatic metastasis are attributed to hsa-mir-577 expression^28^. Next one, hsa-mir-190b, has previously been reported to be upregulated in ER+ breast cancers^29^. Recent findings reveal that hsa-mir-129-1 is linked to triple-negative breast cancer (TNBC) progression^30^; another TNBC biomarker is hsa-mir-522, which regulates cell proliferation, migration, and invasion capacities^31^. On a similar note, hsa-mir-30a controls TNBC aggressiveness^32^. As determined with the Cancer Genome Atlas dataset, hsa-mir-3662 expression is higher in TNBC^33^. Finally, hsa-mir-934 is a potential regulator of the epithelial-mesenchymal transition in TNBC^34^. It has been demonstrated that overexpression of hsa-mir-216a plays a significant role in breast cancer progression and metastasis by leading to the release of pro-inflammatory molecules; hsa-mir-216a, importantly, regulates the crosstalk between cancer cells and the microenvironment cells^35^. Regarding the basal subtype, two miRNAs have been identified, namely 135b and 365, with a central role in regulating the altered functional pathways^36^, and we observe in Fig. 3 that one of them, namely hsa-mir-135b, is influential using our method. The *SPP1* gene plays a significant role in breast cancer by promoting tumor growth, migration, and immune evasion; furthermore, it has been shown that the downregulation of hsa-mir-944 promoted the high expression of *SPP1*, promoting breast cancer cell proliferation^37^. Expression of hsa-mir-133b targets *SOX9* to control pathogenesis and metastasis of breast cancer^38^.

Reviewing those miRNAs with medium importance in Fig. 3, hsa-mir-135a has been shown to be highly expressed in metastatic breast tumors; the expression is required for the migration and invasion of breast cancer cells, but not their proliferation. *HOXA10*, which encodes a transcription factor required for embryonic development and is a metastasis suppressor in breast cancer, was shown to be a direct target of hsa-mir-135 in breast cancer cells^36^. Next, we see hsa-mir-204 that is recently reported as a potential therapeutic target and prognostic marker in breast cancer, emphasizing the need for further research to fully elucidate its complex roles in orchestrating aggressive breast cancer^39^. The high expression of hsa-mir-9-3p affects tumor growth through targeting *MTDH* gene^40^; as shown in Fig. 3, our results show the non-mature miRNA, i.e., hsa-mir-9-3. Findings in a recent work^41^ highlighted that *LRP8* accelerated breast cancer development by contributing to cellular aggressiveness, which was modulated by hsa-mir-1262. Slightly less influential in Fig. 3 is hsa-mir-31, which is shown to promote mammary stem cell expansion and breast tumorigenesis by suppressing Wnt signaling antagonists^42^. Next, hsa-mir-18b promotes breast cancer invasion and metastasis by regulating *TCEAL7*^43^, and there is higher expression of hsa-mir-17-5p in the noninvasive breast cancer cell lines^44^. Finally, hsa-miR-145, appearing in the figure, has been shown to be of importance regarding bone metastasis; recent studies have shown a high probability of interaction among three genes—*c-MYC*, *Cyclin D1*, and *RUNX2*—in bone metastasis, and hsa-miR-145 has been shown to directly target the *MYC* gene^44^.

Finally, among the miRNAs with lower influence scores in Fig. 3 but still high relevance in our analysis, we identify hsa-miR-126, hsa-miR-100, and hsa-let-7a-1, which function as tumor suppressors^44,45^. We also observe hsa-miR-155, a well-known oncogenic miRNA^46^, and hsa-miR-429, which is involved in a regulatory network governing proliferation, migration, and invasion in TNBC cells^47^.

Interestingly, we found that the newly identified miRNAs reported in panel c) in Fig. 3 are primarily associated with various types of neoplasms across different organs. Detailed descriptions of these associations are provided in Supplementary Subsection 2.5.

When averaging the absolute values (magnitude of the importance) over each omic, we notice that the miRNA omic seems to influence the most on the neighboring and similarities of tumor samples. In this section, we provided a feature-based interpretation of OMIDIENT regarding the similarity of the neighboring tumors. In the next section, we provide an omic-based interpretation to complement this section, targeting the cancer subtyping task, rather than highlighting the similarities between neighboring tumors.

#### Interpretation of Omic-type importances

In this section, we look deeper into the contributions of all four components of the latent representations on our performance metrics. In particular, we compute NMI and k-NN accuracy only using different omic-specific or shared representations, as well as their combinations. In other words, we drop the information relative to some omics, and observe the consequent drop (or increase) in performance. This allows us to understand whether the information relevant to the subtyping task is present in all of the omics or concentrated within a specific subset. The result of those ablation studies on both MOCSS and our model is given in Supplementary Subsection 2.6.

As shown in Supplementary Subsection 2.6, we observe that, for all four datasets, MOCSS (AE) reaches either its top or near-top performance with only a single omic available (mRNA for brca, coad, and kirc, and miRNA for lihc). This suggests that the information necessary for subtyping was not effectively disentangled between the shared and omic-specific sub-representations, with each omic-specific representation largely retaining the information from its respective modality. Our model, on the other hand, shows a clear improvement in predictive performance when using the shared sub-representation. While the easier datasets (lihc and kirc) show a similar behavior to MOCSS, with a relatively high performance regardless of the subset of representations used, the other two exhibit their best (or close-to-best) performance either when all specific representations are present, or when the shared representation is used.

In order to formalize these observations, we display the average relative influence that each omic has on the performance. This value attribution is computed through the Shapley value^48^, a well-used method in explainable machine learning^49^.

The Shapley value analysis for all sub-representations, presented in Supplementary Subsection 2.6, confirms that in MOCSS (AE), the shared representation plays a dominant role only for the COAD dataset, and even there only marginally. In contrast, our approach shows that the shared representation is substantially more influential than the three modality-specific representations in three out of the four datasets. We interpret this as evidence that our shared representation is more expressive than that of MOCSS, enabling more effective extraction and integration of information across the different omics modalities.

Finally, OMIDIENT, our proposed method, unlike MOCSS (AE), clearly uses different components of the whole representation for different purposes, i.e., classification and reconstruction. This is evidenced by our ablation study, which shows that OMIDIENT primarily relies on the shared representation for classification. The most informative signals across omics are concentrated in this shared part, consistent with the concept of shared latent spaces in multimodal integration^12^. Meanwhile, due to the architectural design of OMIDIENT, the reconstruction of each omic is handled independently through its omic-specific representation, ensuring that modality-specific details are preserved.

## Discussion

The advancement of omics technologies has improved personalized medicine, particularly transforming cancer research and treatment through more precise diagnosis, subtyping, and therapy selection. Despite the availability of more data annotations than before, multi-omics integration methods that require these labels (supervised approaches) lose the ability to discover new phenotypes or traits (e.g., disease diagnosis, grading of tumors, and cancer subtypes) since they become trained for certain pattern recognition introduced by the labels. To this end, we proposed OMIDIENT, an unsupervised (without using labels) multi-omics integration framework that is capable of finding novel molecular or clinical subtypes. OMIDIENT is based on deep generative models, where we learn comprehensive embeddings representing both omic-specific and omic-shared information of multi-omics data. This compressed and informative representation can subsequently be used for various downstream tasks such as cancer subtyping. OMIDIENT also demonstrates strong capability in reconstructing missing values across omics datasets. To summarize, OMIDIENT is a multi-omics representation learning framework based on deep generative models with superior performance in typical use-cases compared to other state-of-the-art unsupervised approaches.

Unlike similar generative models, OMIDIENT can capture structured biological or functional relationships by dividing omic features into distinct groups. The use of groups enables the separation of embeddings, yielding a more structured and modular representation of multi-omics data that captures its inherent complexity. While we only demonstrated the capability of OMIDIENT with respect to subtyping, classification, data reconstruction, and limited biological interpretability, OMIDIENT allows for improving the expressiveness and potential interpretability of the learned representations due to its notion of feature grouping. Further investigation can be conducted to identify explicit groups within the learned embeddings to infer gene sets or any phenotypic categories. As such, the contribution of each biological module can be analyzed both independently and in combination.

Despite the fact that we utilized mRNA expression, DNA methylation, and miRNA expression data for the multi-omics integration in this paper, the framework can be easily extended to accommodate additional data types. For the shared latent representation, extending the model to additional modalities only requires invoking the contrastive learning module in a pairwise manner. This does not introduce any architectural or conceptual changes; the implementation consists of a few additional lines of code to call the module and update the loss accordingly. For the omic-specific components, the existing network architecture can be reused, and incorporating a new modality simply requires adding its reconstruction and KL-divergence terms, again amounting to only minor code additions. We note that different omics may differ in scale, sparsity, and noise characteristics. While the current framework is directly reusable for other data types, modality-specific architectures may further improve performance in some cases; for example, copy number variation data may benefit from a sequential neural network design. Even in such cases, the required modifications remain minimal and localized.

In addition to biological interpretations for breast and colorectal cancer, we have provided two orthogonal interpretability analyses of our method. First, Shapley value analysis quantifies the global contribution of each omic modality to subtype discrimination, revealing the dominant role of the shared embedding. Second, gradient-based analysis shows that, at the feature level, miRNA signals consistently drive local neighborhood structure in the latent space. Together, these findings indicate that while global performance relies on balanced multimodal integration, local similarity relationships can be disproportionately shaped by miRNA features without necessarily affecting overall classification outcomes.

For future work, additional priors could be incorporated into the framework to further enhance the model. Moreover, OMIDIENT can be extended to other omics modalities as well as longitudinal data.

## Methods

### Framework

The deep learning architecture in OMIDIENT builds upon that of MOCSS^7^, with the shared goal of extracting both omic-specific and omic-shared information from multi-omics data. While MOCSS employs standard autoencoders to learn modality-specific features, OMIDIENT leverages a novel (to the best of our knowledge) type of variational autoencoder framework, enabling it to model uncertainty and structure the latent space to better capture distinct tumor characteristics. We use a product of Dirichlet distribution to model the latent representations instead of the deterministic assignment of latent representations using a mere autoencoder; more details can be found in the next section, Probabilistic Graphical Model. For the omic-shared part, we use contrastive learning as in MOCSS; however, we do not use the loss function that MOCSS introduced. Namely, the orthogonal loss is not used in OMIDIENT to disentangle the omic-specific and omic-shared information. This choice is supported by our ablation study. As a result, we have three loss functions: reconstruction loss, contrastive loss, and our proposed loss for the product of a Dirichlet variational autoencoder, which we explain in the next section.

### Probabilistic graphical model

A standard variational autoencoder (VAE)^17^ learns a posterior distribution *q*_*ϕ*_(*Z*∣*X*) (short form *q*(*Z*)) through a neural network, assuming that the prior distribution is $p(Z)={{{\mathcal{N}}}}(0,I)$.

Subsequently, other models have been proposed to model the prior as Dirichlet, Dirichlet Variational Autoencoders (DirVae) with various approximations; one method approximated Dirichlet by Laplace approximation^18^. Here we refer to it as LapDirVae, as shown in Eq. (1). A more recent method approximates Dirichlet samples by a Gamma distribution, instead of a Gaussian, by computing the inverse of the CDF of the Gamma distribution^11^:

1 $$\begin{array}{l}\,q(Z)=Dir(Z;{\alpha }_{1:K})\approx {{{\rm{Softmax}}}}(X),\,{{{\rm{where}}}}\\ \quad {\mu }_{k}=\log {\alpha }_{k}-\frac{1}{K}\sum\limits_{k}{\alpha }_{k},\hfill \\ \quad {\sigma }_{k}=\frac{1}{{\alpha }_{k}}\left(1-\frac{2}{K}\right)+\frac{1}{{K}^{2}}\sum\limits_{k}\frac{1}{{\alpha }_{k}},\,{{{\rm{and}}}}\hfill \\ \quad {\!} {X}_{k}={\mu }_{k}+\epsilon {\sigma }_{k},\,\epsilon \sim {{{\mathcal{N}}}}(0,1).\hfill\end{array}$$

We propose *ProdDirVae*, a tangent of a variational autoencoder that is governed by a product of *S* Dirichlet priors. Let *p*(*Z*) denote the prior, and let it follow *S* independent Dirichlet distributions, as shown in Eq. (2). Note that *S* is the number of groups into which the representations are decomposed. Moreover, *K* is the dimensionality of each group of representations, which represents the dimensionality of the Dirichlet distribution.

2 $$p(Z)={\prod }_{s=1}^{S}Dir\left({Z}^{s};{({\alpha }_{1}^{0})}^{s},\ldots ,{({\alpha }_{K}^{0})}^{s}\right)$$

The motivation of this prior is threefold: (1) the Dirichlet prior models the latent variable as a vector of categorical probabilities as opposed to the Gaussian distribution which assumes a smooth, unimodal distribution, not necessarily valid for tumors that may deviate nonsmoothly between individuals; (2) the product of Dirichlet decomposes the latent space into independent parts, i.e., enabling it to represent a variety of unrelated characteristics of a tumor; (3) Moreover, we fix the concentration parameters to be 0.5, which promotes sparsity of the tumor characteristics. This is the default Jeffreys prior for the Dirichlet distribution with parameters ${\alpha }_{k}^{0}=0.5$.

We learn the posterior *q*(*Z*) from the encoder of the neural network. The posterior also takes the form of a product of a Dirichlet distribution. For sampling the Dirichlet, we use the reparameterization trick by computing the inverse of the CDF of Gamma distribution as in Dirichlet VAE^11^, here we name it GamDirVae. Therefore, we have the following form shown in Eq. (3); for details of the approximation and all the graphical model illustrations, see Supplementary Section 1.

3 $$\begin{array}{c}q(Z)={\prod }_{s=1}^{S}{Dir}({Z}^{s};{\alpha }_{1}^{s},\ldots ,{\alpha }_{K}^{s}),\\ {Z}^{s}\sim {Dir}({\alpha }_{1}^{s},\ldots ,{\alpha }_{K}^{s})\,\hat{=}\,{\hat{Z}}_{k}^{s}={(u{\alpha }_{k}^{s}{\rm\Gamma }({\alpha }_{k}^{s}))}^{\frac{1}{{\alpha }_{k}^{s}}},\\ s.t. \; \; u \sim {Uniform}(0,1).\\ {Z}_{k}^{s}=\frac{{\hat{Z}}_{k}^{s}}{{\sum }_{k}{\hat{Z}}_{k}^{s}}\end{array}$$

Given a prior Dirichlet distribution with parameters for each group *s*, ${({\alpha }^{0})}_{k}^{s}=0.5$—the Jeffreys prior—the Kullback–Leibler (KL) divergence between this prior and the approximate posterior with parameters ${\alpha }_{k}^{s}$ serves as the KL loss term in our model and is formulated as follows.

4 $${{loss}}_{{KL}}={\sum}_{s}{\sum}_{k}{{{\mathrm{log}}}}{\rm\Gamma }({({\alpha }^{0})}_{k}^{s})-{{{\mathrm{log}}}}{\rm\Gamma }({\alpha }_{k}^{s})+({\alpha }_{k}^{s}-{({\alpha }^{0})}_{k}^{s})\psi ({\alpha }_{k}^{s})$$

### Encoder and decoder

In this section, we present the innovative components of our neural network architecture, i.e., the encoder and decoder for each omic modality. The encoder transforms the input omic data *X* into *S* groups of ${\hat{\alpha }}_{1:K}$. Subsequently, to ensure these parameters meet the positivity requirement of the Dirichlet distribution, i.e., positive concentration parameters, we apply a sigmoid activation function on them, resulting in concentration parameters *α*_1:*K*_. Using the concentration parameters and Gamma approximation trick, explained in Supplementary Section 1, the omic representation *Z* is computed. Our decoder, more detailed in Supplementary Section 1, begins by applying a softmax activation function to the temperature-scaled latent variable *Z*, i.e., *τ*^−1^*Z* with *τ* = ∑_*k*_*Z*_*k*_. The scaling of the variable *Z* is intended to counteract the normalization effect inherent in the computation of the Dirichlet variable, allowing the model to instead take advantage of a sharper discrimination provided by the softmax function^50^. The output of the softmax function serves as the basis for all the results reported in this paper. Ablation studies empirically demonstrate that this design choice leads to improved performance.

### Shapley values

In order to formalize method interpretation, we display the average relative influence that each omic has on the performance. This value attribution is computed through the Shapley value^48^, a well-used method in explainable machine learning^49^. Formally, assuming *n*_*o*_ omic-types, $E=\{{e}_{1},...,{e}_{{n}_{o}+1}\}$ the set of sub-representations (one for each omic, and one shared). Then, we call $v:{2}^{E}\mapsto {\mathbb{R}}$, the set function that returns the value of a metric (k-NN accuracy, K-Means NMI) when using only representation dimensions included in the set. In practice, the influence of sub-representation *e*_*i*_ on metric *v* is computed as:

5 $${\phi }_{{e}_{i}}(v)={\sum}_{S\subseteq E\setminus \{{e}_{i}\}}\frac{| S| !(| E| -| S| -1)!}{| E| !}(v({S}\cup \{{e}_{i}\})-v(S)),$$

which is the average of the marginal contribution of *e*_*i*_ to each possible coalition of sub-representations. Since we have only four sub-representations (∣*E*∣ = 4), it is feasible to use the Shapley value even if it has exponential complexity.

### Setting

Table S16, in Supplementary Subsection 4.1, summarizes the key hyperparameters used in our method (OMIDIENT) and the neural network based benchmarks. These include the shapes of the neural network layers, batch size, and the temperature parameter, which controls the model’s sensitivity to hard samples. It also lists the tuning hyperparameters: learning rate, weight decay, and the number of groups *S*, which determines the level of structured representation in our model.

### Dataset

We investigate the capacity of our method, OMIDIENT, with regard to representation learning by applying it to data from five different cancers: breast cancer (BRCA), colon adenocarcinoma cancer (COAD), colorectal cancer (CRC), liver cancer (LIHC), and kidney cancer KIR of type clear renal cell carcinoma (KIRC). The COAD, LIHC, and KIRC datasets are based upon data generated by The Cancer Genome Atlas (https://www.cancer.gov/tcga). The BRCA dataset obtained from MOCSS^7^ has labels indicating five subtypes within breast cancer, i.e., luminal A, luminal B, normal-like, basal-like, and HER2-enriched^51,52^, each of which is classified by PAM50, a 50-gene signature^6^. The COAD dataset has labels indicating four subtypes within colon cancer, i.e., CIN, GS, microsatellite instability (MSI), and POLE^53^. Microsatellite instability (MSI) arises through defective DNA mismatch repair in the tumors, causing mutations particularly at repetitive DNA sequences called “microsatellites.” The LIHC dataset contains both cancer and normal samples^54^, and the KIRC dataset has two subgroups^55^. The CRC data were described in ref. ^56^, and the published subset included the first 103 tumor-normal pairs, and the rest of the sample pairs were collected and processed in the same manner. CRC data has two types of annotations, one with four classes and another with two classes. The MSI classification system is relevant for CRC prognosis and treatment^19^. For the CRC with four classes, we used the following groups: (1) microsatellite instability (MSI), (2) microsatellite stability (MSS), (3) ambiguous tumors, and (4) normal samples. For the CRC with consensus molecular subtypes (CMS) grouping^26^, we used the following groups: (1) CM1 group containing microsatellite instability immune, 14%, hyper-mutated, microsatellite unstable, and strong immune activation, and (2) other CMS groups. The set of CRC samples was described in ref. ^56^. The published subset included the first 103 tumor-normal pairs, and the rest of the sample pairs were collected and processed in the same manner.

For all datasets except CRC, three types of omics data, i.e., mRNA expression data (mRNA), DNA methylation data (meth), and miRNA expression data (miRNA), are used to provide integrative and complementary information on each cancer dataset. For CRC, mRNA and DNA methylation are available, and the top 10000 features with the largest variance in each omic are chosen. The BRCA dataset is preprocessed with noise reduction^6^, which results in a more refined list of omic features. Feature engineering is not applied to the rest of the datasets. The information on the number of features in each omic data for each cancer and the number of cancer samples is listed in Table S17 in Supplementary Subsection 4.2. For class imbalance regarding the five datasets, see Fig. S20 in Supplementary Subsection 4.2.

### Statistics and reproducibility

We performed the statistical analysis by training neural networks and chose the learned representations corresponding to a model with the highest ELBO, i.e., the lowest validation loss function. For the performance metrics, we used diverse tools such as NMI, F1-score, AUC-ROC, and Spearman and Pearson for correlation analysis. For K-means results in terms of NMI, we repeated K-means 30 times across all data and methods, then picked the best-performing one. For k-NN classification, we selected the smallest *k* that achieved comparable performance to larger *k* values, favoring parsimony and reducing the risk of overfitting to local structure. To assess the statistical significance of classification performance under severe class imbalance in the CRC dataset, we additionally conducted permutation testing, which provides a non-parametric significance estimate robust to distributional assumptions. All experiments are reproducible according to the reported data in Sections Data availability and Code availability, which have the predefined seeds that are used for reproducibility.

### Reporting summary

Further information on research design is available in the Nature Portfolio Reporting Summary linked to this article.

## Supplementary information

Supplementary Information

Description of Additional Supplementary Files

Supplementary Data 1

Supplementary Data 2

Supplementary Data 3

Supplementary Data 4

Supplementary Data 5

Reporting Summary

## Data Availability

The BRCA, COAD, LIHC, and KIRC datasets are based upon data generated by The Cancer Genome Atlas (https://www.cancer.gov/tcga). We obtained the preprocessed BRCA and COAD datasets from https://github.com/ChenyuxinXMU/MOCSSand https://github.com/GaoLabXDU/MultiOmicsIntegrationStudy/, respectively. For LIHC and KIRC cancers, we obtained them from https://acgt.cs.tau.ac.il/multi_omic_benchmark/download.html. The CRC dataset is deposited in the European Genome-phenome Archive (EGA, https://www.ebi.ac.uk/ega/) for controlled access; the study accession number is EGAS00001000854. For source data relating to the plots in the supplementary file and main figures, see Suppl_Data1.xlsx, Suppl_Data2.odg, Suppl_Data3.txt, Suppl_Data4.txt, and Suppl_Data5.txt.

## Code availability

The source code of this work can be downloaded from https://github.com/negar7918/multiomics and https://zenodo.org/records/20273710.
