# Supplementary material for: OMIDIENT: Multiomics Integration for Cancer by Dirichlet Auto-Encoder Networks
Source: Commun Biol. 2026 Jul 6;9:1416. doi: 10.1038/s42003-026-10578-1 (PMC13720109; doi:10.1038/s42003-026-10578-1)
Supplement: Supplementary file 1 [file 42003_2026_10578_MOESM1_ESM.pdf]

OMIDIENT: Multiomics Integration for Cancer by Dirichlet Auto-Encoder Networks

Negar Safinianaini[1*], Niko Välimäki[2,3], Roman Bresson[4], Alexandra Gorbonos[1], Kristiina Rajamäki[2,3], Lauri A. Aaltonen[2,3,5], Pekka Marttinen[1]

[1*]Dept. of Computer Science, Aalto University, Helsinki, Finland.
[2]Dept. of Medical and Clinical Genetics, University of Helsinki, Helsinki, Finland.
[3]Applied Tumor Genomics Research Program, Research Programs Unit, University of Helsinki, Helsinki, Finland.
[4]Mohamed bin Zayed University of Artificial Intelligence, Abu Dhabi, UAE.
[5]iCAN Digital Precision Cancer Medicine Flagship, University of Helsinki, Helsinki, Finland.

*Corresponding author(s). E-mail(s): negar.safinianaini@aalto.fi;

1 Dirichlet Representations

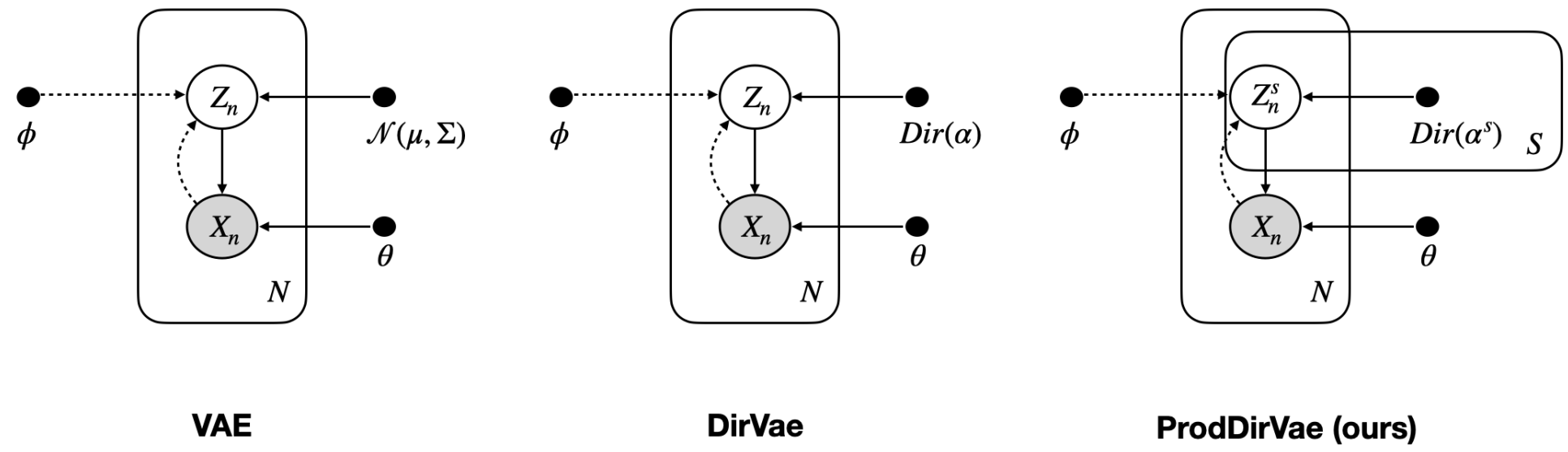

Fig. S1: The graphical models of VAE, Dirichlet VAE (DirVae) and Product of Dirichlet VAE, ProdDirVae (our model) are shown.

In this section, we review the details for the reparameterization trick in modeling the latent representation with a Dirichlet distribution [1]; this is also useful for our model: the product of Dirichlet distributions. To see the difference in the modelling of method compared to DirVAE and VAE see Fig. S1.

Firstly, we know that a Dirichlet distributed values with K dimensions can be formulated as K normalized Gamma distributions, i.e., $Z_k \sim Gam(\alpha_k, 1)$ and $normalized(Z_1, \ldots, Z_K) \sim Dir(\alpha_1, \ldots, \alpha_K)$. Ffor sampling Gamma values, we use the Inverse CDF Method, or Probability Integral Transform, which states that for any random variable Z with its CDF, $F(Z)$, we can sample Z by first sampling $u \sim Unif(0,1)$, and then computing $Z = F^{-1}(u)$ [2]. Given $Z_k = F^{-1}_{\alpha_k,1}(u)$, i.e., $u = F_{\alpha_k,1}(Z_k)$, we write the approximated Gamma CDF for small values of Z_k—since these will be normalized for the Dirichlet samples we are not constrained to small values in the final step.

$$F_{\alpha_k,1}(Z_k) = \frac{1}{\Gamma(\alpha_k)}\gamma(\alpha_k, Z_k) \approx \frac{Z_k^{\alpha_k}}{\alpha_k\Gamma(\alpha_k)} \quad \text{corresponds to} \quad Z_k^{\alpha_k} = \alpha_k\Gamma(\alpha_k)F_{\alpha_k,1}(Z_k)$$

Computing the inverse of the above, we achieve

$$Z_k = \Big(F_{\alpha_k,1}(Z_k)\alpha_k\Gamma(\alpha_k)\Big)^{1/\alpha_k} = \Big(u\alpha_k\Gamma(\alpha_k)\Big)^{1/\alpha_k}.$$

Letting C denote $u\alpha\Gamma(\alpha)$, Figure S2 illustrates the final reparameterization in the Dirichlet networks. Note that we use sigmoid to ensure α vector of concentration parameters stays positive, and we normalize the sampled Gamma values so that the samples have the Dirichlet distribution.

Our decoder, shown in Fig. S3, begins by applying a softmax activation function to the temperature-scaled latent variable Z, i.e., $\tau^{-1}Z$ with $\tau = \sum_k Z_k$. The scaling of the variable Z is intended to counteract the normalization effect inherent in the

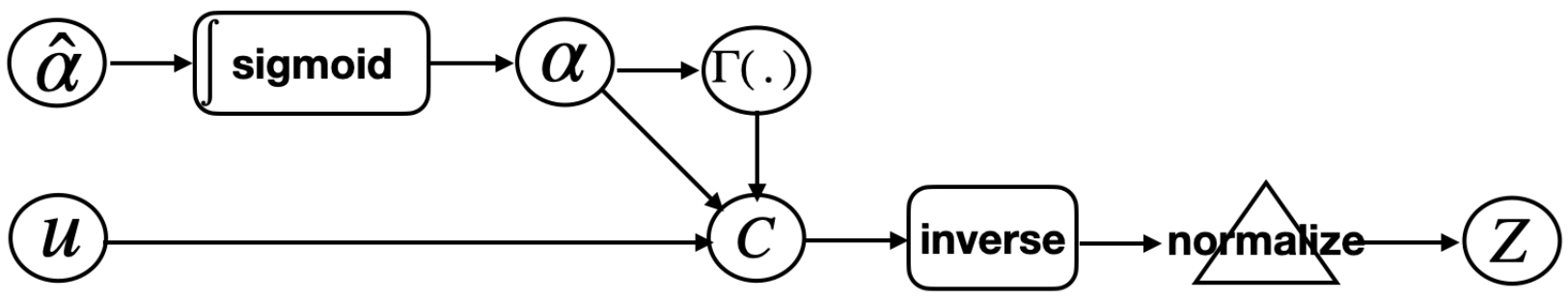

Fig. S2: The Dirichlet Reparameterization of variable Z.

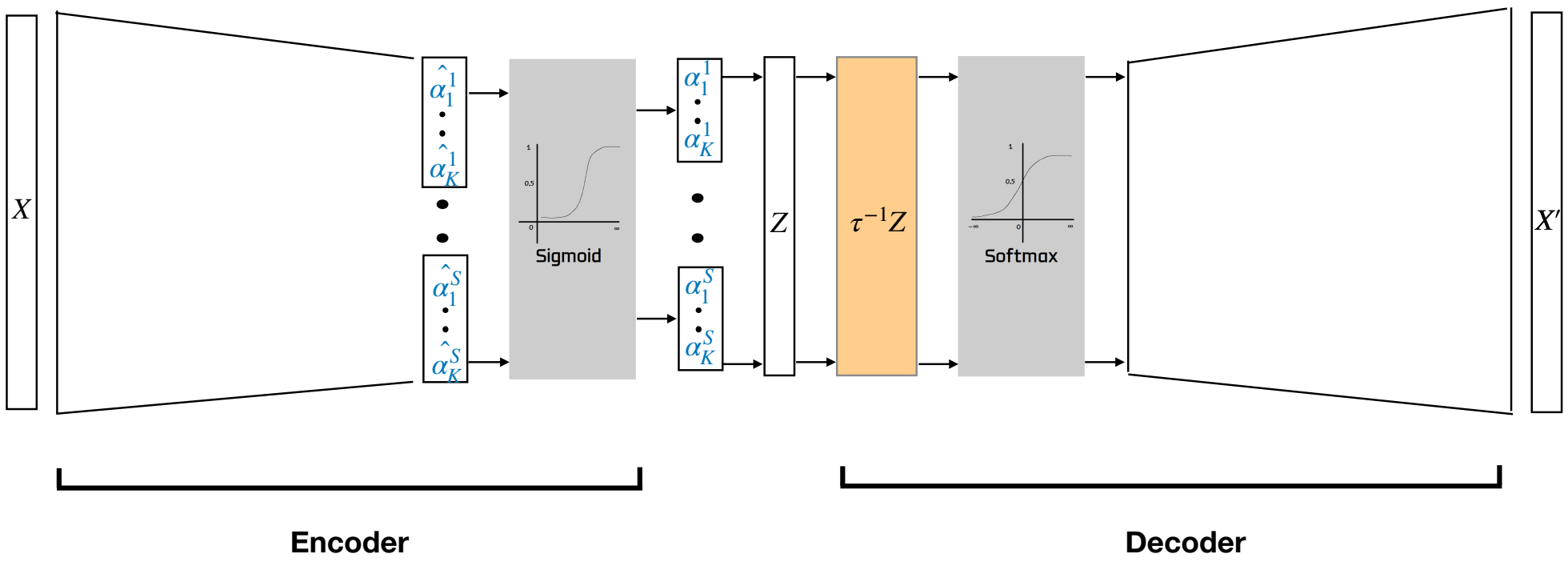

Fig. S3: The overview of OMIDIENT's encoder and decoder architecture.

computation of the Dirichlet variable, allowing the model to instead take advantage of a sharper discrimination provided by the softmax function [3]. The output of the softmax function serves as the basis for all the results reported in this paper. Ablation studies empirically demonstrate that this design choice leads to improved performance.

2 Model Explainability

2.1 UMAP Illustration of CRC Samples Regarding MSI

In Fig. S4, we compare the UMAP illustration of the samples' learned representations by the top-performing models in clustering and classification, i.e., MOCCS (AE), VAE, LapDirVae, and OMIDIENT (ours). The samples include both tumors and normal colon samples, i.e., entirely nonneoplastic tissue. The axes x and y of each plot correspond to the two components of the UMAP transformation.

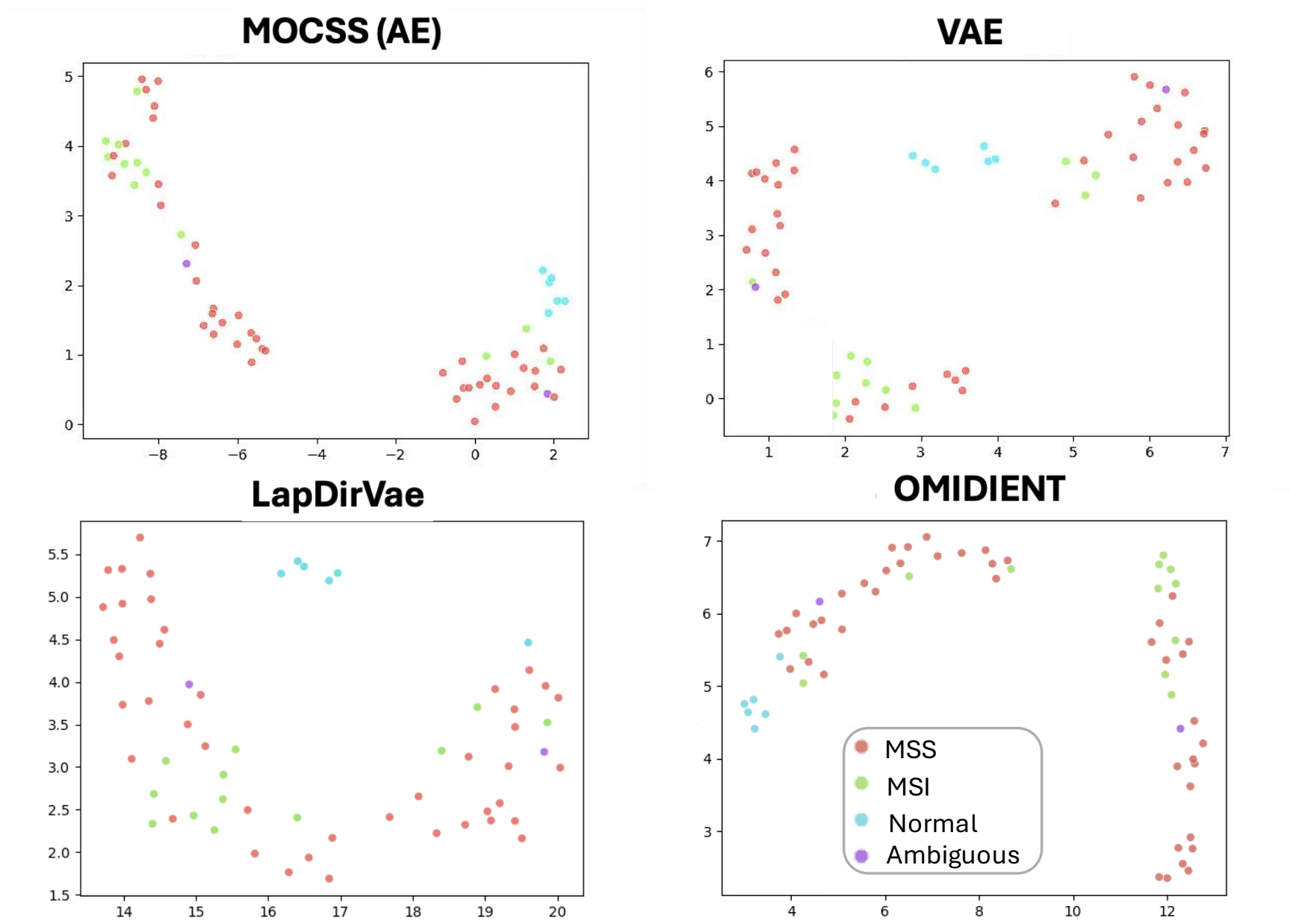

Fig. S4: The samples' learned representations by MOCSS (AE), VAE, LapDirVae, and Ours (OMIDIENT), which are mapped to UMAP, are shown.

2.2 UMAP Illustration of CRC Samples Regarding CMS

In Fig. S5, we present UMAP visualizations of the learned representations from the top-performing models in clustering and classification, namely MOCSS (AE), LapDirVae, MultiVI, and our method, OMIDIENT. Our method achieves the highest NMI score, OMIDIENT, demonstrating a more distinct and well-separated grouping of CMS1 samples (highlighted in red) in the top-right region of the plot. In contrast, MOCSS places the CMS1 samples in close proximity to, or overlapping with, other CMS groups, indicating less discriminative representations. Finally, although MultiVI and LapDirVae achieve high accuracy, the UMAP plots reveal poor clustering of CMS1 (highlighted in red), suggesting that the seemingly high accuracy may be an artifact caused by class imbalance. This is further supported by its low NMI score. In Fig. S6, CMS2-4 are not well classified across different methods.

2.3 UMAP Illustration of CRC Samples Regarding Gender and Stage

We annotate the samples by gender and tumor stages in Fig. S7. We can observe that our method distinguishes the genders clearly. Note that, due to the use of UMAP for dimensionality reduction, not all learned dimensions are visualized. As a result, some female samples appear on the left-hand side (LHS), representing normal, non-tumor tissue. Comparing this plot to Fig. S4, we can observe that "normal-adjacent" (male) tumor cluster in OMIDIENT has fewer MSI tumors and the "normal-distant" (female)

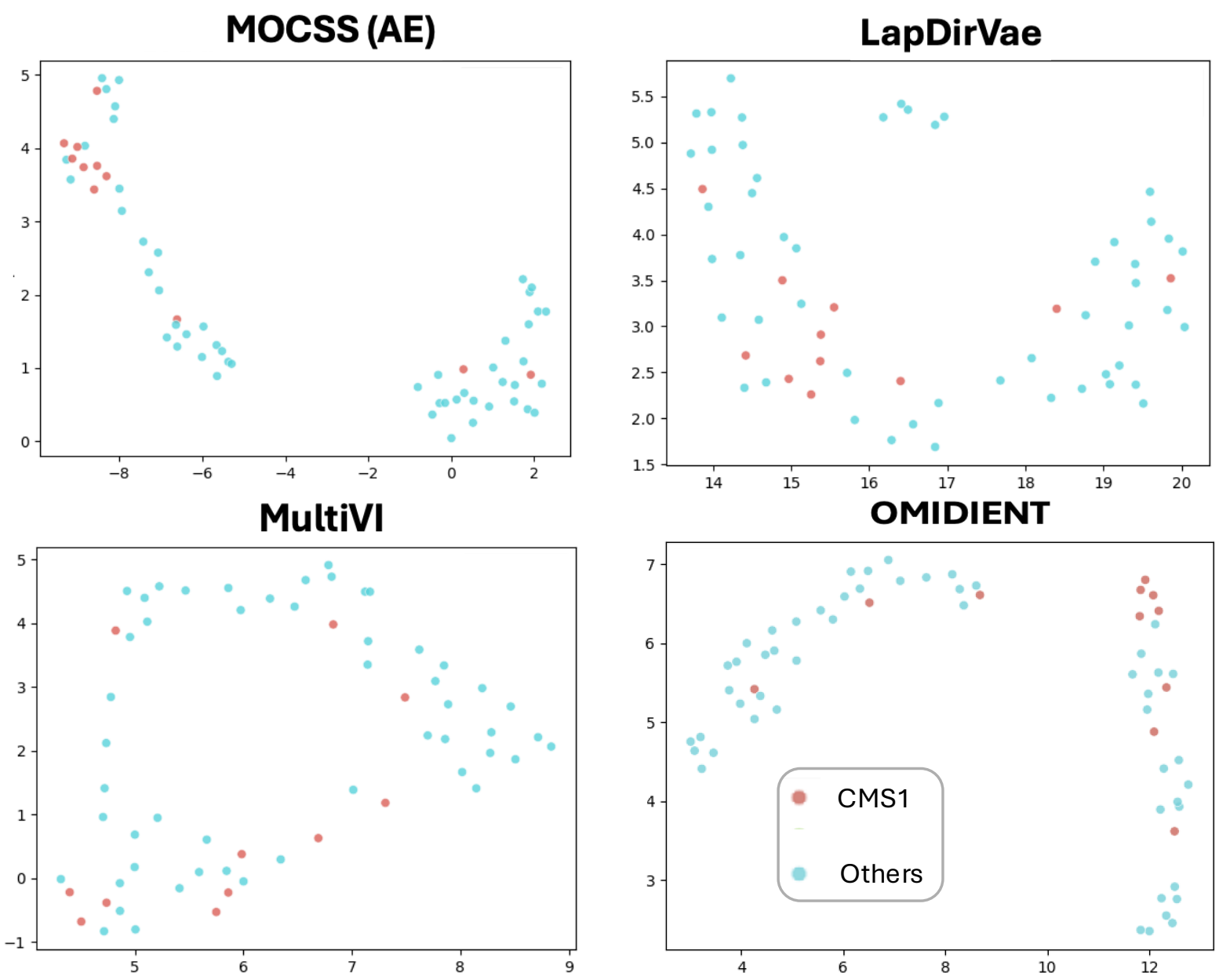

Fig. S5: The samples' learned representations by MOCSS (AE), LapDirVAE, MultiVI, and OMIDIENT, mapped onto UMAP, are shown. The first two rows compare CMS1 versus the other subtypes; CMS1 tumors, highlighted in red, are most distinctly clustered in OMIDIENT, consistent with the NMI results reported in Table **??**.

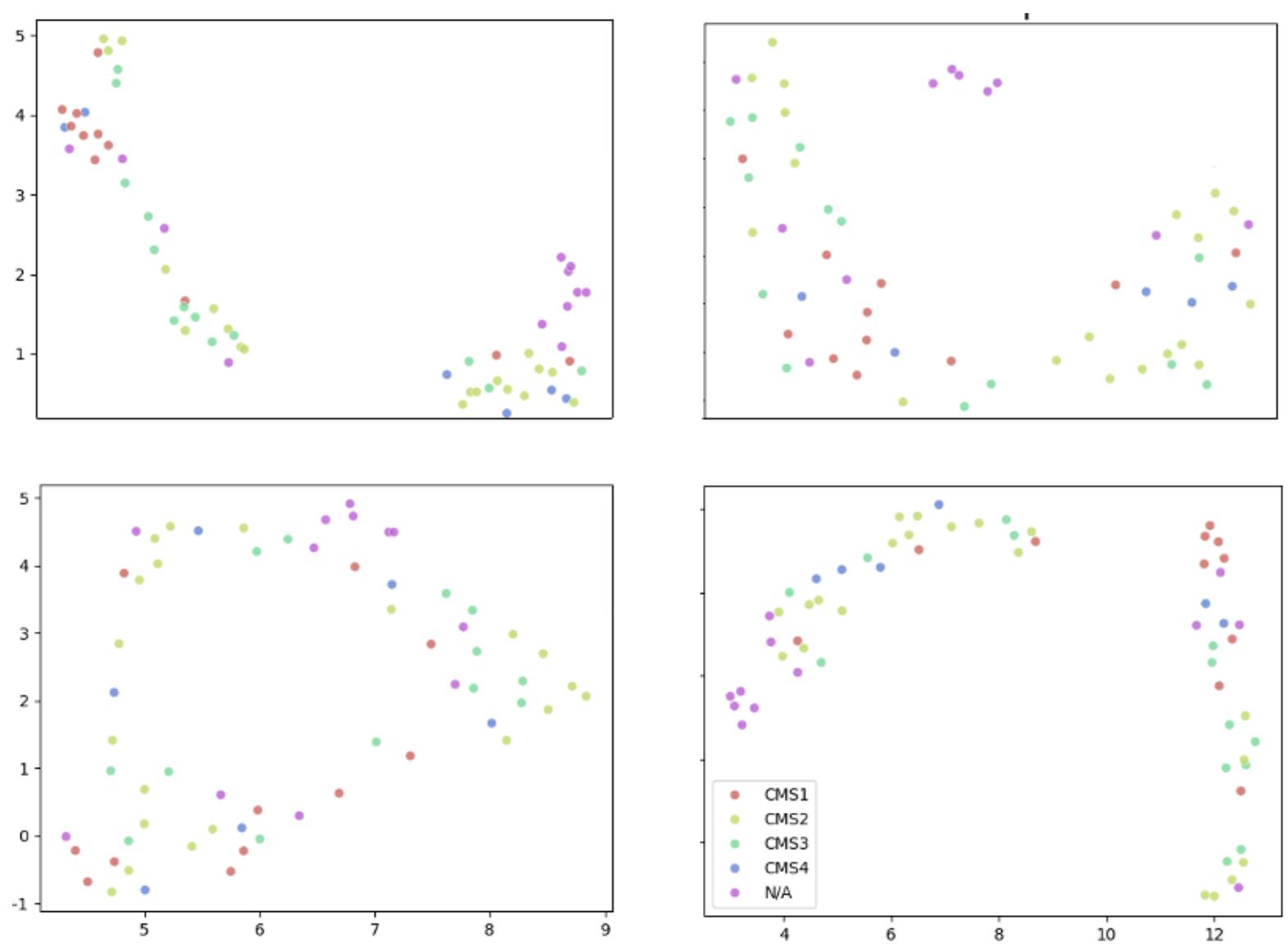

Fig. S6: The figures display the full CMS labelings of Fig. S5 for MOCSS (AE), LapDirVAE, MultiVI, and OMIDIENT, arranged from top left to bottom right.

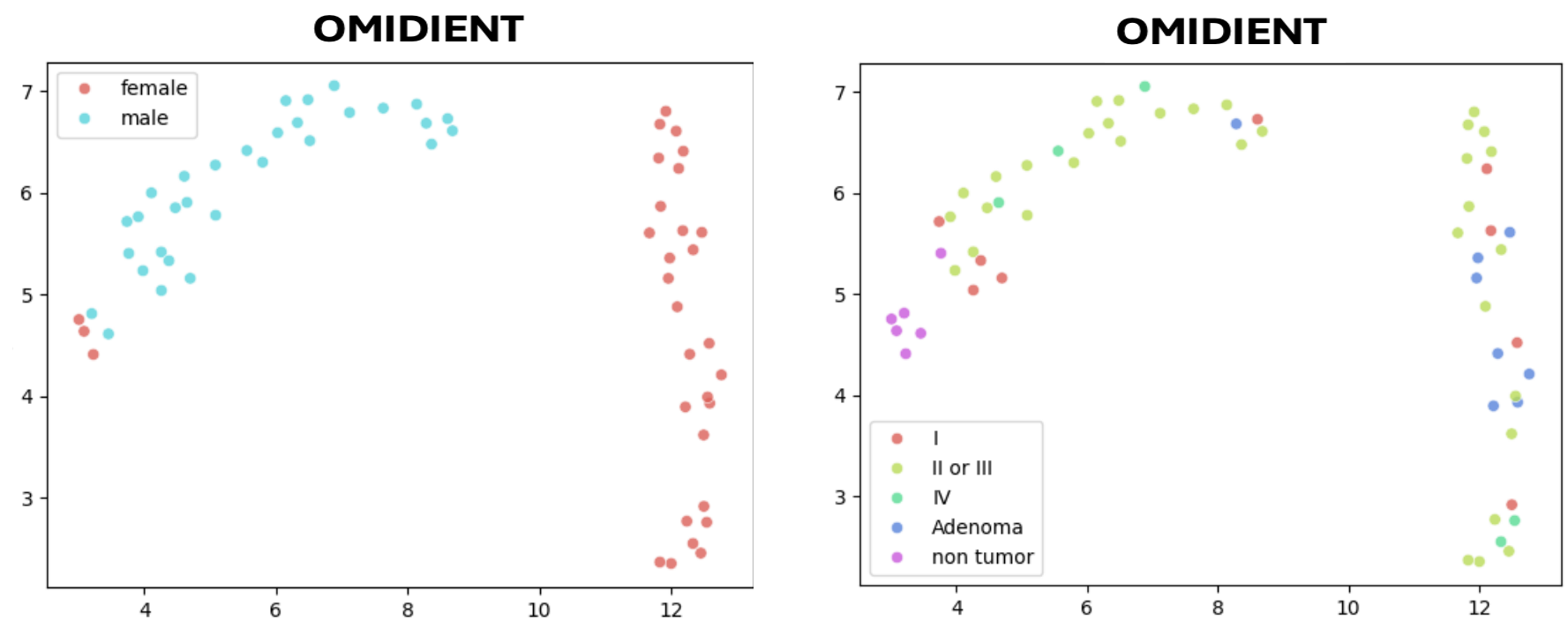

Fig. S7: The samples' learned representations by OMIDIENT. In the LHS, the gender is annotated. In the RHS, tumor stages are annotated, where non-tumor refers to normal samples and Adenoma to benign tumor samples.

tumor cluster has more MSI tumors. This observation is consistent with the well-established finding that MSI is more prevalent in colorectal cancers (CRCs) among females than males. It also appears that in Fig. S4, OMIDIENT can distinguish the MSI subgroup among women quite well into its own half of the female cluster, while in the male cluster MSI tumors are fully scattered around. In the figure on the right side, the tumor stages are annotated and the Adenomas are clustered in the middle of the right cluster, being close to stages I and II, which is sensible.

We removed the batch effect. The clustering and classification results were similar to those reported earlier, and the new plots are shown in Fig. S8.

2.4 UMAP Illustration of BRCA with Different Stages

As shown in Fig. S9, the tumor stages are annotated on breast cancer samples via UMAP projection of the raw data and embeddings gained by OMIDIENT. The stages are very imbalanced, e.g. there are only two samples with stage 2. If we look closer, we see that the stages are quite mixed in the raw data plot, while in each cluster detected by OMIDIENT, the stages are more separated. For example, the cluster in the top middle part of the RHS plot, shows red (stage 1) and blue (stage 3) points separated. Given the heterogeneity of breast cancer, it is expected to have a mix of stages in the plot. By comparing the frequencies of stages in the clusters, we observe that the top right cluster has the most density of high stages tumors, stage 3 and 4, that is 64% and 25%, respectively.

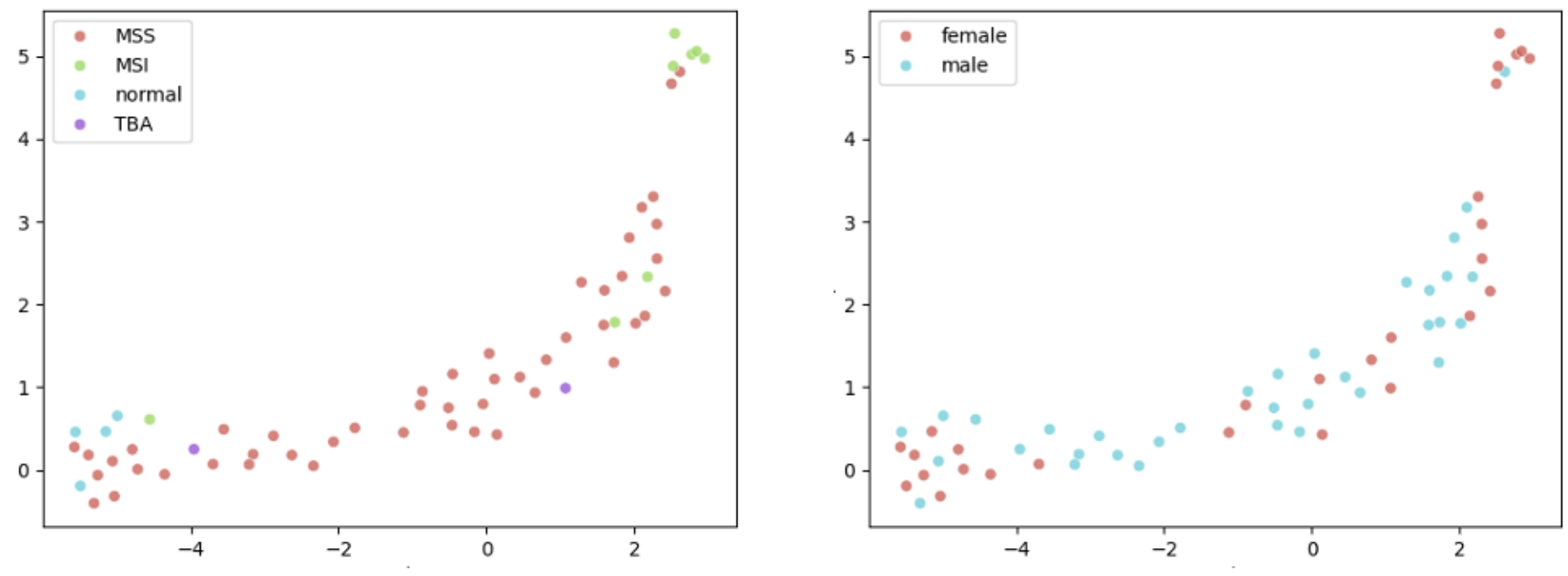

Fig. S8: After batch removal effect, the samples' learned representations by OMIDIENT are shown. In the LHS, we see the annotation with regard to MSI and MSS labels, and on the RHS, we can see that the genders are no longer separated due to the batch removal effect.

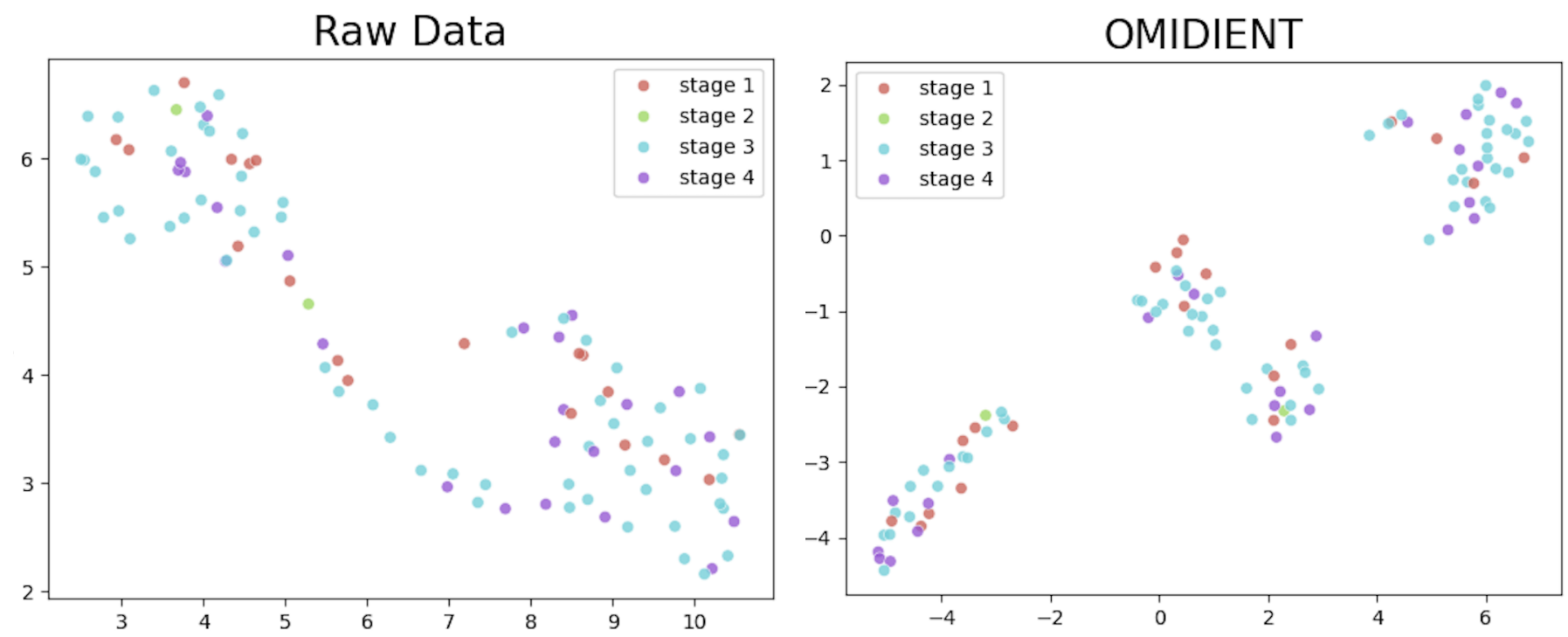

Fig. S9: The tumor stages are shown on the UMAP projection of BRCA raw data and embeddings from OMIDIENT, in LHS and RHS, respectively.

2.5 Analysis of Local Sensitivity of BRCA Tumors

As shown in Fig. S10, OMIDIENT identifies the top miRNA biomarkers in BRCA, with those highlighted in red corresponding to biomarkers previously reported in the state of the art. Notably, a substantial fraction of the top biomarkers detected by OMIDIENT have been clinically validated in prior studies, supporting the biological relevance of our findings.

Additionally, looking into Human MiRNA Disease Database version 3 [4], we have found associations between newly (to the best of our knowledge) detected miRNA biomarkers, that is mainly neoplasms. These are shown in Table S1, to S14.

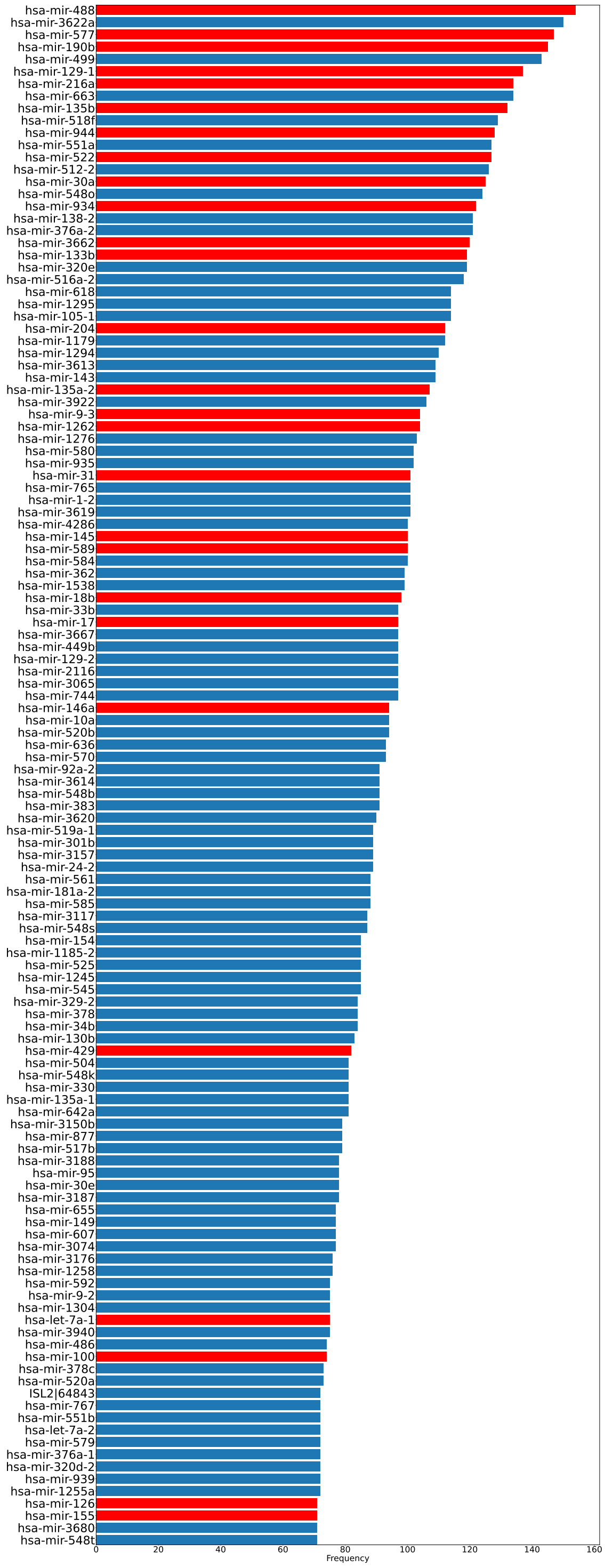

Fig. S10: The top frequent miRNAs for breast cancer; the red ones are established by s-o-t-a.

mirna	evidence code	disease
hsa-mir-499	genetics GWAS	Breast Neoplasms
hsa-mir-499	other	Stomach Neoplasms
hsa-mir-499	genetics GWAS	Esophageal Neoplasms
hsa-mir-499	circulation biomarker diagnosis ns	Genital Neoplasms, Female
hsa-mir-499	genetics GWAS	Uterine Cervical Neoplasms
hsa-mir-499	exosome	Endometrial Neoplasms
hsa-mir-499	therapeutic target	Lung Neoplasms

Table S1: Associations of hsa-mir-499

mirna	evidence code	disease
hsa-mir-663	genetics knock down promote	Ovarian Neoplasms
hsa-mir-663	genetics knock down promote	Lung Neoplasms
hsa-mir-663	epigenetics	Skin Neoplasms
hsa-mir-663	genetics overexpression promote	Urinary Bladder Neoplasms
hsa-mir-663	other	Colorectal Neoplasms
hsa-mir-663	epigenetics	Pancreatic Neoplasms
hsa-mir-663	genetics overexpression promote	Stomach Neoplasms

Table S2: Associations of hsa-mir-663

mirna	evidence code	disease
hsa-mir-3622a	lncRNA target	Colorectal Neoplasms
hsa-mir-518f	genetics overexpression suppress	Breast Neoplasms
hsa-mir-518f	other	Colorectal Neoplasms
hsa-mir-518f	transcription factor target	Prostatic Neoplasms
hsa-mir-518f	tissue expression ns	Stomach Neoplasms
hsa-mir-551a	genetics overexpression promote	Breast Neoplasms
hsa-mir-551a	genetics GWAS	Colorectal Neoplasms
hsa-mir-551a	target gene	Stomach Neoplasms
hsa-mir-551a	other	Ovarian epithelial cancer
hsa-mir-512-2	tissue expression ns	Urinary Bladder Neoplasms
hsa-mir-548o	therapeutic target	Breast Neoplasms

Table S3: Associations of hsa-mir-3622a, 518f, 551a, 512-2, and 548o.

mirna	evidence code	disease
hsa-mir-138-2	other	Urinary Bladder Neoplasms
hsa-mir-138-2	other	Head and Neck Neoplasms
hsa-mir-138-2	target gene	Nasopharyngeal Neoplasms
hsa-mir-138-2	target gene	Lung Neoplasms
hsa-mir-138-2	target gene	Ovarian Neoplasms
hsa-mir-138-2	target gene	Neoplasms
hsa-mir-138-2	tissue expression down	Thyroid Neoplasms

Table S4: Associations of hsa-mir-138-2

mirna	evidence code	disease
hsa-mir-320e	target gene	Breast Neoplasms
hsa-mir-320e	circulation biomarker diagnosis up	Esophageal Neoplasms
hsa-mir-320e	other	Colorectal Neoplasms
hsa-mir-516a-2	lncRNA target	Urinary Bladder Neoplasms
hsa-mir-516a-2	other	Breast Neoplasms
hsa-mir-516a-2	target gene	Ovarian Neoplasms
hsa-mir-516a-2	therapeutic target	Stomach Neoplasms
hsa-mir-618	genetics GWAS	Breast Neoplasms
hsa-mir-618	genetics GWAS	Colorectal Neoplasms
hsa-mir-618	other	Stomach Neoplasms
hsa-mir-618	other	Thyroid Neoplasms
hsa-mir-618	target gene	Prostatic Neoplasms

Table S5: Associations of hsa-mir-320e, 516a-2, and 618.

mirna	evidence code	disease
hsa-mir-1294	target gene	Uterine Cervical Neoplasms
hsa-mir-1294	tissue expression down	Stomach Neoplasms
hsa-mir-1294	therapeutic target	Breast Neoplasms
hsa-mir-1294	genetics knock down promote	Stomach Neoplasms
hsa-mir-3613	tissue expression ns	Ovarian epithelial cancer
hsa-mir-3613	tissue expression ns	Breast Neoplasms
hsa-mir-3613	tissue expression down	Colonic Neoplasms
hsa-mir-1276	genetics overexpression promote	Colonic Neoplasms
hsa-mir-1276	circRNA target	Ovarian Neoplasms
hsa-mir-1276	lncRNA target	Breast Neoplasms
hsa-mir-1276	lncRNA target	Stomach Neoplasms
hsa-mir-1276	tissue expression ns	Laryngeal Neoplasms
hsa-mir-1276	other	Urinary Bladder Neoplasms

Table S6: Associations of hsa-mir-1294, 3613, 1276

mirna	evidence code	disease
hsa-mir-580	exosome	Breast Neoplasms
hsa-mir-580	therapeutic target	Stomach Neoplasms
hsa-mir-580	other	Ovarian Neoplasms

Table S7: Associations of hsa-mir-580

mirna	evidence code	disease
hsa-mir-33b	other	Stomach Neoplasms
hsa-mir-33b	target gene	Breast Neoplasms
hsa-mir-33b	target gene	Lung Neoplasms
hsa-mir-33b	target gene	Ovarian Neoplasms
hsa-mir-33b	genetics overexpression suppress	Colorectal Neoplasms
hsa-mir-4286	circulation biomarker diagnosis ns	Stomach Neoplasms
hsa-mir-4286	other	Prostatic Neoplasms
hsa-mir-4286	target gene	Lung Neoplasms
hsa-mir-4286	tissue expression ns	Colorectal Neoplasms

Table S8: Associations of hsa-mir-33b and 4286.

mirna	evidence code	disease
hsa-mir-1179	genetics knock down promote	Stomach Neoplasms
hsa-mir-1179	genetics overexpression promote	Breast Neoplasms
hsa-mir-1179	genetics knock down promote	Gallbladder Neoplasms
hsa-mir-1179	genetics overexpression promote	Mouth Neoplasms
hsa-mir-1179	genetics knock down suppress	Uterine Cervical Neoplasms
hsa-mir-1179	circRNA target	Thyroid Cancer, Papillary
hsa-mir-1179	lncRNA target	Ovarian Neoplasms
hsa-mir-1179	tissue expression ns	Prostatic Neoplasms
hsa-mir-1179	tissue expression up	Colorectal Neoplasms

Table S9: Associations of hsa-mir-1179

mirna	evidence code	disease
hsa-mir-765	genetics overexpression promote	Stomach Neoplasms
hsa-mir-765	other	Gallbladder Neoplasms
hsa-mir-765	genetics overexpression promote	Colonic Neoplasms
hsa-mir-765	other	Colorectal Neoplasms
hsa-mir-765	other	Central Nervous System Neoplasms
hsa-mir-765	tissue expression up	Rectal Neoplasms
hsa-mir-765	genetics knock down promote	Breast Neoplasms
hsa-mir-765	genetics overexpression suppress	Endometrial Neoplasms
hsa-mir-765	genetics knock down promote	Ovarian Neoplasms

Table S10: Associations of hsa-mir-765

mirna	evidence code	disease
hsa-mir-92a-2	other	Urinary Bladder Neoplasms
hsa-mir-92a-2	circulation biomarker diagnosis down	Breast Neoplasms
hsa-mir-92a-2	circulation biomarker diagnosis ns	Colorectal Neoplasms
hsa-mir-92a-2	other	Esophageal Neoplasms
hsa-mir-92a-2	other	Ovarian Neoplasms
hsa-mir-92a-2	tissue expression up	Thyroid Neoplasms
hsa-mir-92a-2	exosome	Liver Neoplasms
hsa-mir-519a-1	tissue expression ns	Stomach Neoplasms

Table S11: Associations of hsa-mir-92a-2 and 519a-1

mirna	evidence code	disease
hsa-mir-636	other	Prostatic Neoplasms
hsa-mir-636	genetics overexpression suppress	Uterine Cervical Neoplasms
hsa-mir-636	other	Urinary Bladder Neoplasms
hsa-mir-636	genetics overexpression promote	Cystic Fibrosis
hsa-mir-636	genetics overexpression promote	Ovarian Neoplasms
hsa-mir-636	transcription factor target	Colorectal Neoplasms
hsa-mir-636	lncRNA target	Endometrial Neoplasms
hsa-mir-636	circulation biomarker diagnosis ns	Pancreatic Neoplasms
hsa-mir-636	tissue expression up	Colonic Neoplasms
hsa-mir-636	genetics knock down promote	Lung Neoplasms

Table S12: Associations of hsa-mir-636

mirna	evidence code	disease
hsa-mir-362	genetics overexpression promote	Thyroid Neoplasms
hsa-mir-362	genetics overexpression promote	Triple Negative Breast Neoplasms
hsa-mir-362	epigenetics	Uterine Cervical Neoplasms
hsa-mir-362	other	Stomach Neoplasms
hsa-mir-362	genetics knock down promote	Urinary Bladder Neoplasms
hsa-mir-362	genetics knock down suppress	Ovarian Neoplasms
hsa-mir-362	genetics overexpression promote	Pituitary Neoplasms
hsa-mir-362	genetics overexpression promote	Paranasal Sinus Neoplasms
hsa-mir-362	circulation biomarker diagnosis ns	Colorectal Neoplasms
hsa-mir-362	lncRNA target	Mouth Neoplasms
hsa-mir-362	target gene	Lung Neoplasms
hsa-mir-362	therapeutic target	Kidney Neoplasms
hsa-mir-362	other	Breast Neoplasms

Table S13: Associations of hsa-mir-362

mirna	evidence code	disease
hsa-mir-10a	genetics overexpression promote	Colonic Neoplasms
hsa-mir-10a	genetics overexpression promote	Endometrial Neoplasms
hsa-mir-10a	genetics GWAS	Colorectal Neoplasms
hsa-mir-10a	other	Head and Neck Neoplasms
hsa-mir-10a	other	Laryngeal Neoplasms
hsa-mir-10a	circulation biomarker diagnosis up	Thyroid Cancer, Papillary
hsa-mir-10a	other	Ovarian Neoplasms
hsa-mir-10a	other	Biliary Tract Neoplasms
hsa-mir-10a	other	Kidney Neoplasms
hsa-mir-10a	lncRNA target	Uterine Cervical Neoplasms
hsa-mir-10a	transcription factor target	Breast Neoplasms
hsa-mir-10a	target gene	Pancreatic Neoplasms
hsa-mir-10a	other	Prostatic Neoplasms
hsa-mir-10a	exosome	Stomach Neoplasms
hsa-mir-10a	genetics overexpression promote	Urinary Bladder Neoplasms
hsa-mir-10a	target gene	Thyroid cancer, follicular
hsa-mir-10a	tissue expression up	urinary system cancer
hsa-mir-10a	other	Lung Neoplasms

Table S14: Associations of hsa-mir-10a

2.6 Ablation Studies

Fig. S11 shows that OMIDIENT uses shared embedding compared to MOCSS (AE).

The Shapley values for all sub-representations are provided in Fig. S12. There, we confirm our previous observation, where, in MOCSS, the shared representation plays a preponderant role only on coad, yet only marginally so. On the other hand, in our approach, we see that the shared representation is significantly more influential than the three specific ones on 3 of the 4 datasets. We interpret this as showing that our shared representation is more expressive than MOCSS, achieving powerful extraction and aggregation of the information from each omic.

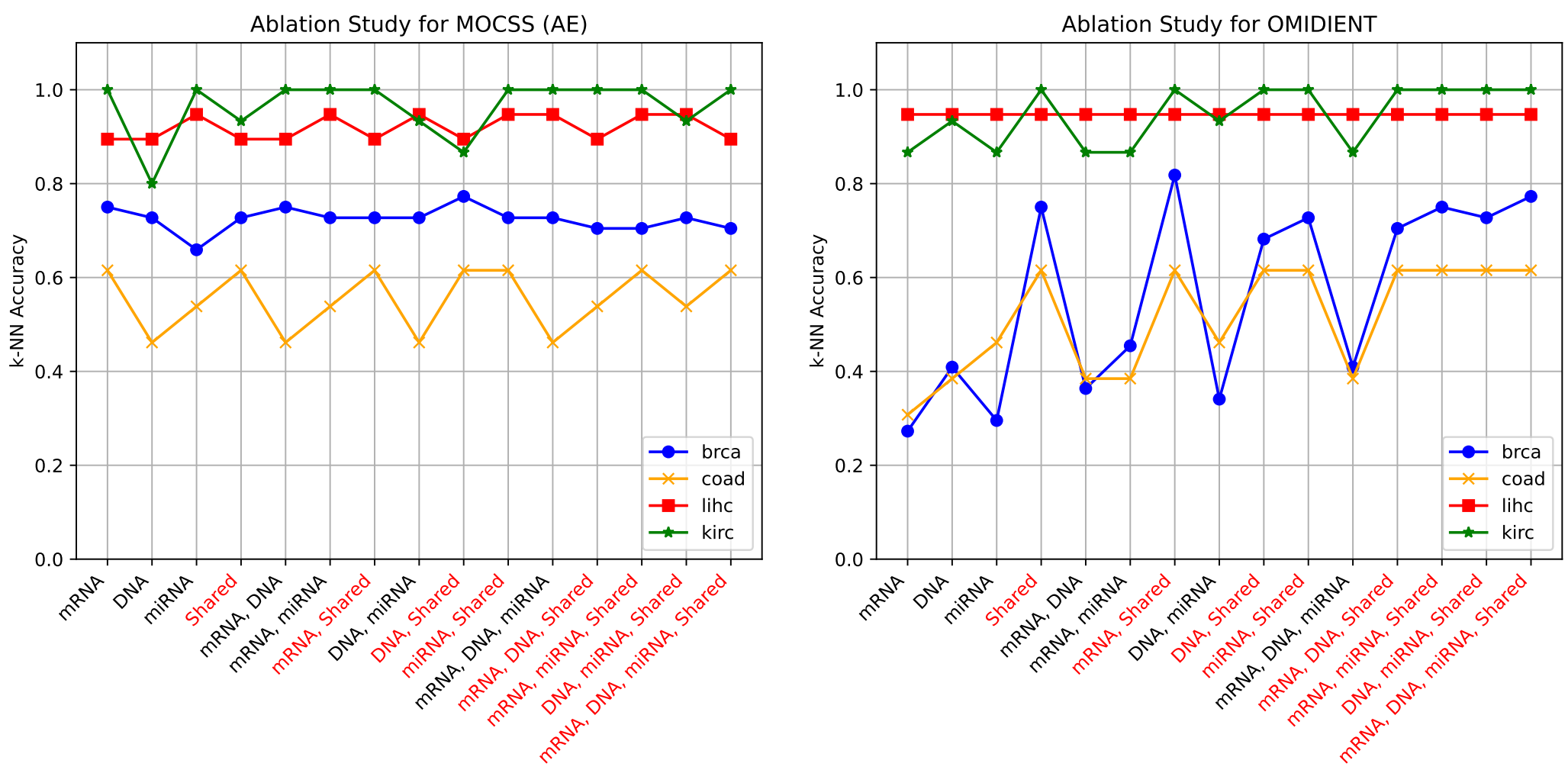

Fig. S11: MOCSS' and our method's ablation study of different omic combinations based on k-NN accuracy. Highlighted values use the shared representation.

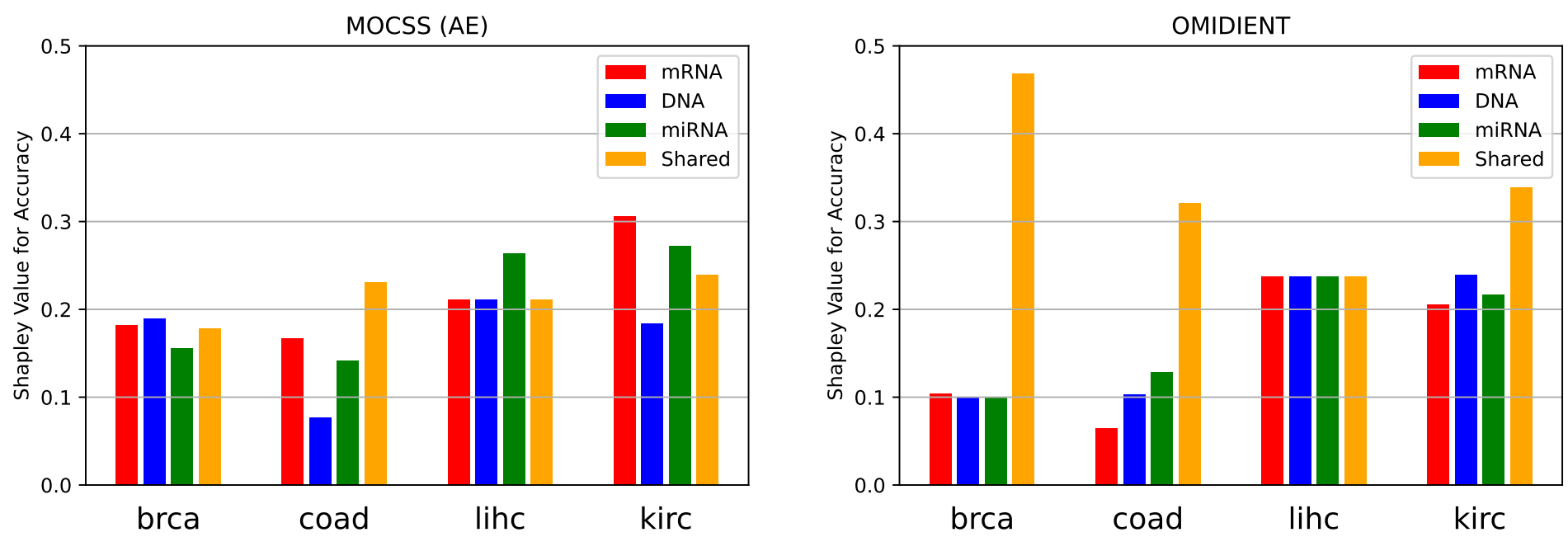

Fig. S12: Shapley values for accuracy of k-NN

Fig. S13 depicts the result of the ablation studies for our model, OMIDIENT. Fig. S14 to S18 depicts the result of the ablation studies for other models and all diseases. Each box plot uses $n = 4$ cancer types: BRCA, COAD, LIHC, and KIRC.

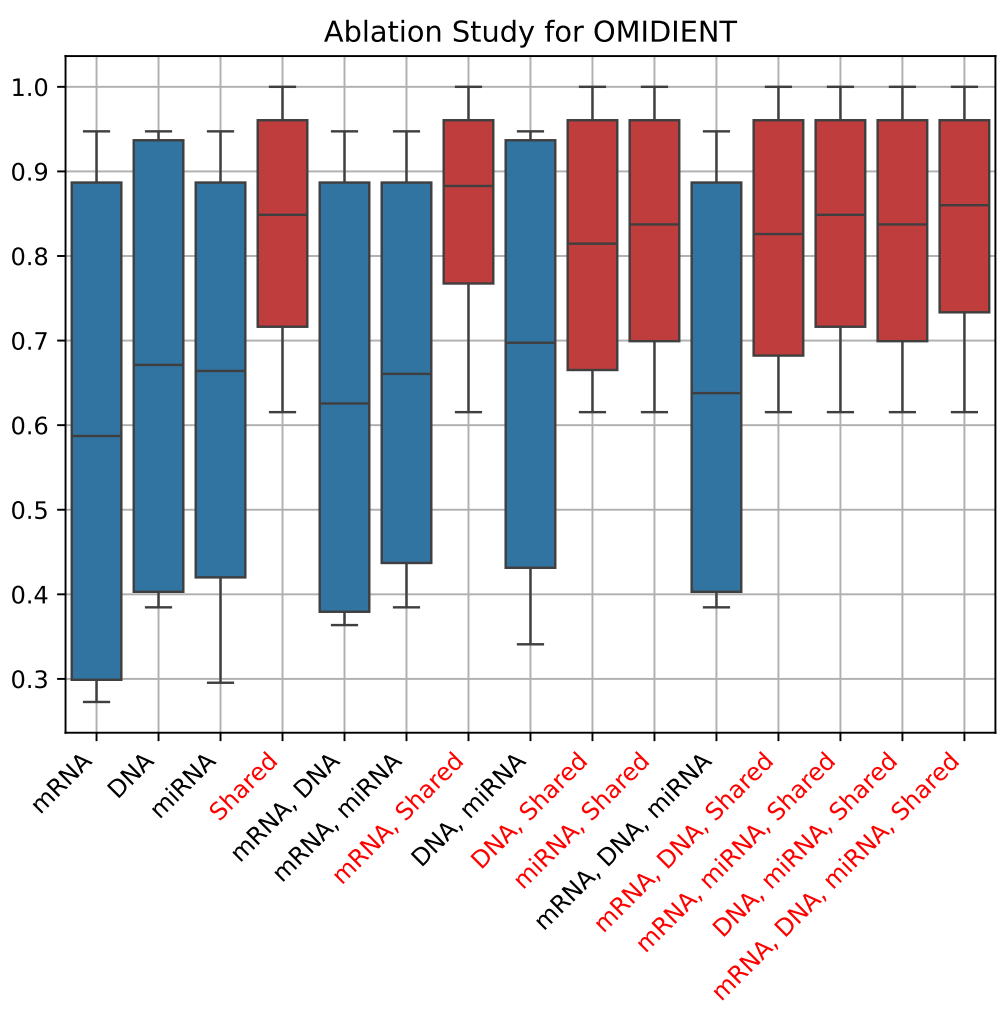

Fig. S13: Boxplot view of figure S11. The red boxes use the shared representation.

2.7 Shapley values

Fig. S19 depicts the Shapley values of the different sub-representations for all models and all diseases.

3 Additional Classification Results

For the data with high class imbalance, that is CRC with two clusters CMS1 versus other CMS types, we computed the AUC-ROC and performed permutation test, shown in Table S15. The permutation based p-value is an unreliable indicator of true significance at this sample size (n=16), as the resolution of the permutation distribution is fundamentally limited by the small number of distinct label arrangements. Nevertheless, the evidence collectively favors MOFA2, i.e., it achieves the highest AUC-ROC score and retains a meaningful p-value under permutation.

4 Setting

4.1 Neural Network Setting

Table S16, summarizes the key hyperparameters used in our method (OMIDIENT) and the neural network based benchmarks. We used fully connected linear layers and applied hyperbolic tangent as activation function of each layer.

4.2 Data Setting

Table S15: AUC and permutation test p-values for CRC data with two clusters.

Method	AUC	Permutation p-value
MOCSS (AE)	0.64	0.69
MOFA2	**0.87**	**0.001**
LassoAE	0.64	0.68
Concerto	0.53	0.57
MultiVI	0.73	0.26
DeFusion	0.46	0.81
VAE	0.60	0.54
LapDirVae	0.71	0.73
GamDirVae	0.67	0.55
OMIDIENT (ours)	0.69	0.37

Table S16: Hyperparameter Setting

Hyperparameter	Setting
Shape of mRNA encoder-decoder layers	{512, 256, 128, 32, 32, 128, 256, 512}
Shape of DNA methylation encoder-decoder layers	{512, 256, 128, 32, 32, 128, 256, 512}
Shape of miRNA encoder-decoder layers	{256, 128, 64, 32, 32, 64, 128, 256}
Learning rate	[.0003, .0002, .0001, .0004, .0005, .0006]
Weight decay	[5e-4, 4e-4, 3e-4, 6e-4, 7e-4]
Number of groups for OMIDIENT	[2, 3, 4, 5]
Dimension of each group for OMIDIENT	8
Batch size	32
Temperature	0.4

Table S17: Summary of datasets with the number of tumor samples and ground-truth classes

Dataset	mRNA expression	miRNA expression	DNA methylation	Samples	Classes
BRCA	1000	503	1000	875	5
COAD	17260	17261	19052	260	4
CRC	10000	N/A	10000	309	4, 2
LIHC	20530	1046	5000	378	2
KIRC	58315	1879	22928	289	2

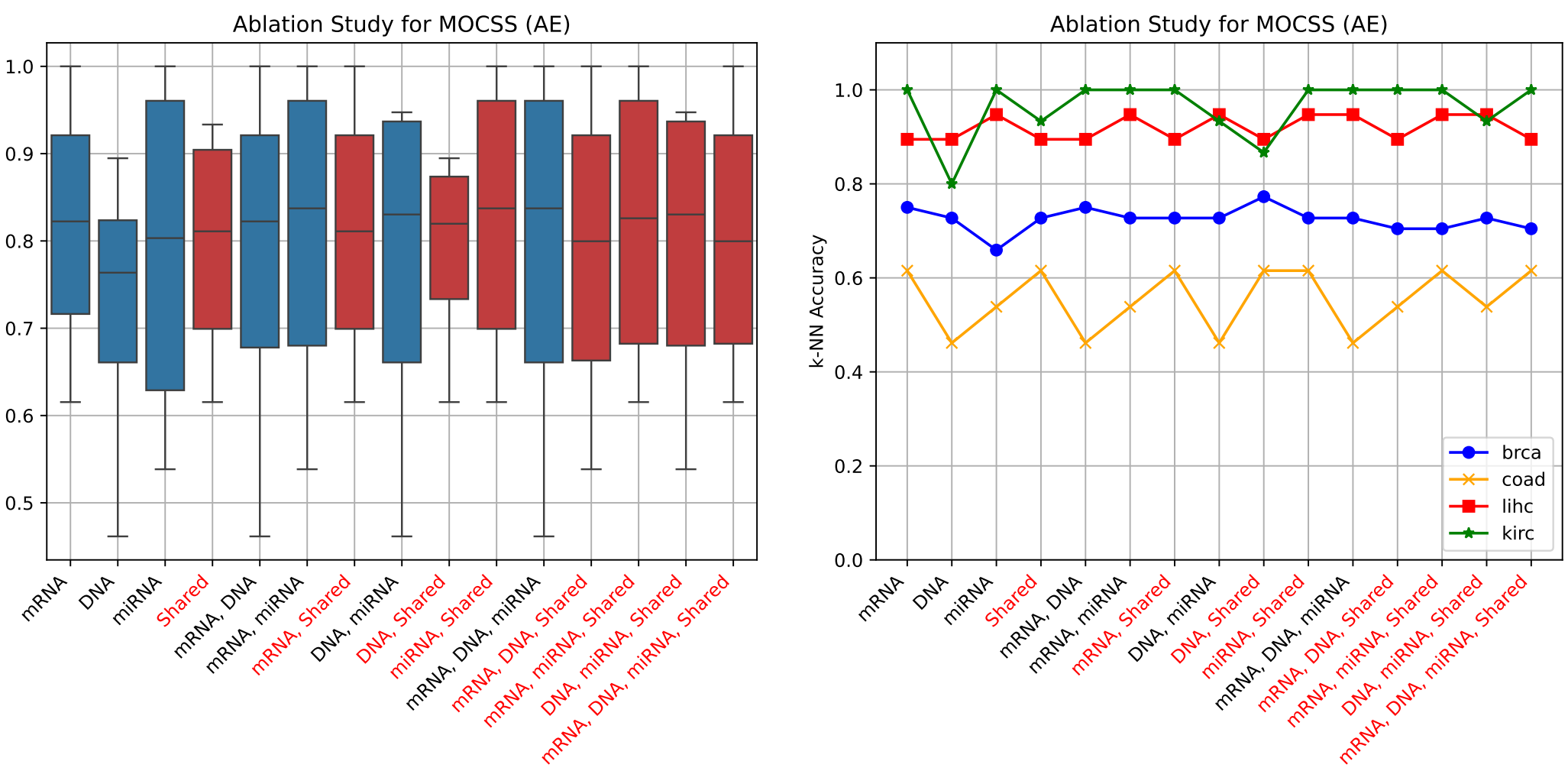

Fig. S14: Ablation results values AE (MOCSS)

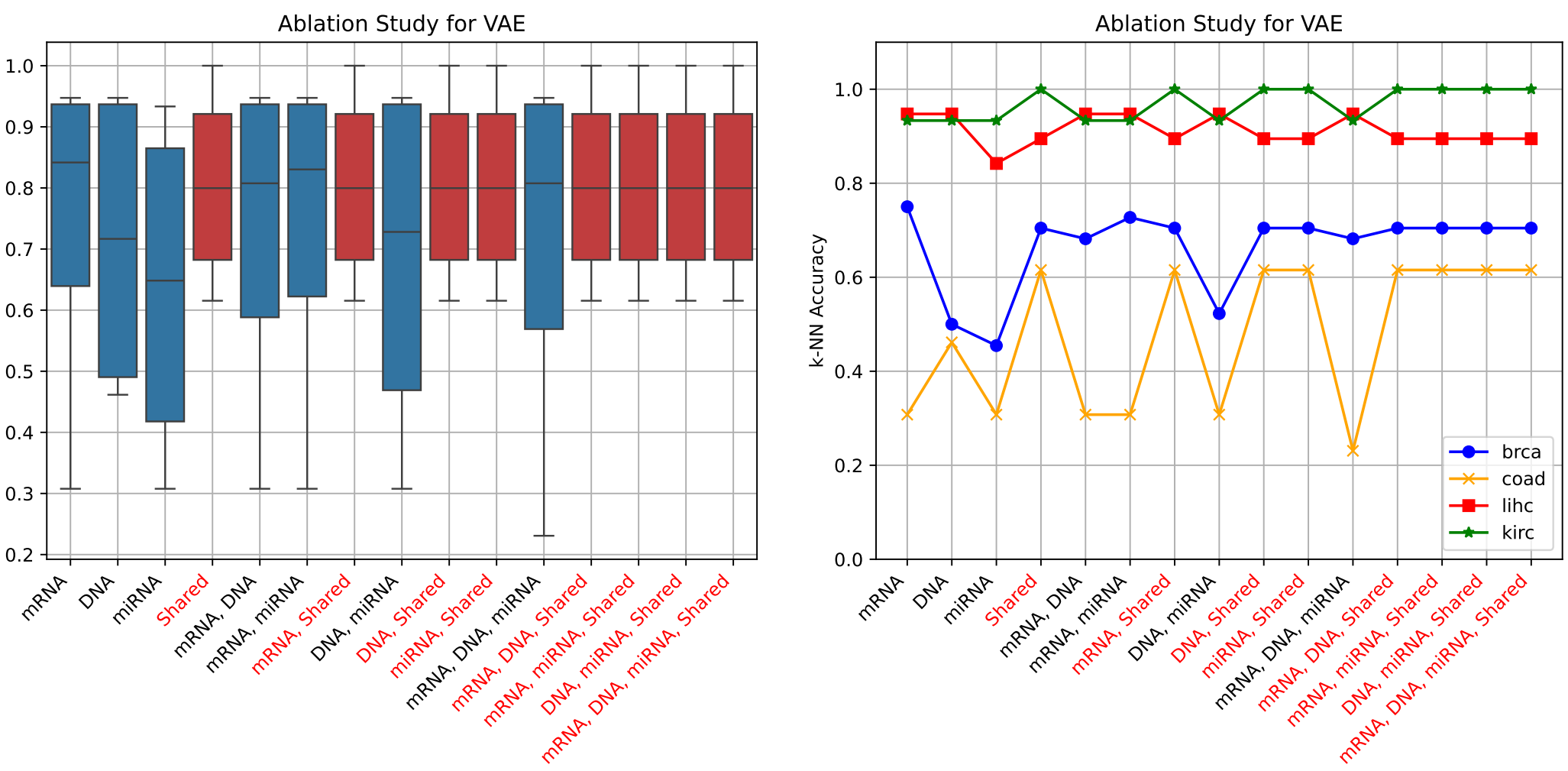

Fig. S15: Ablation results values for accuracy of VAE

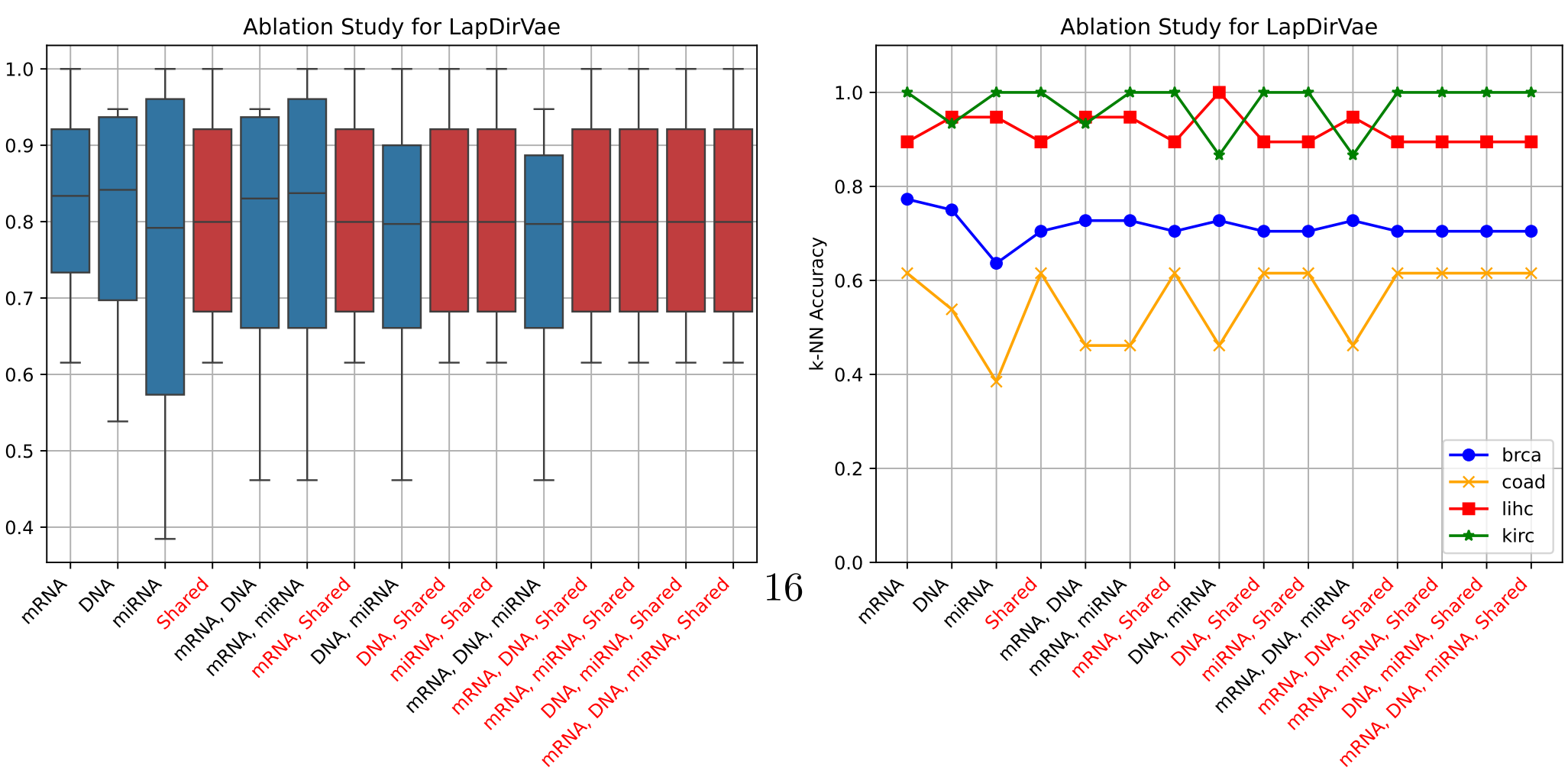

Fig. S16: Ablation results values for accuracy of LapDirVAE

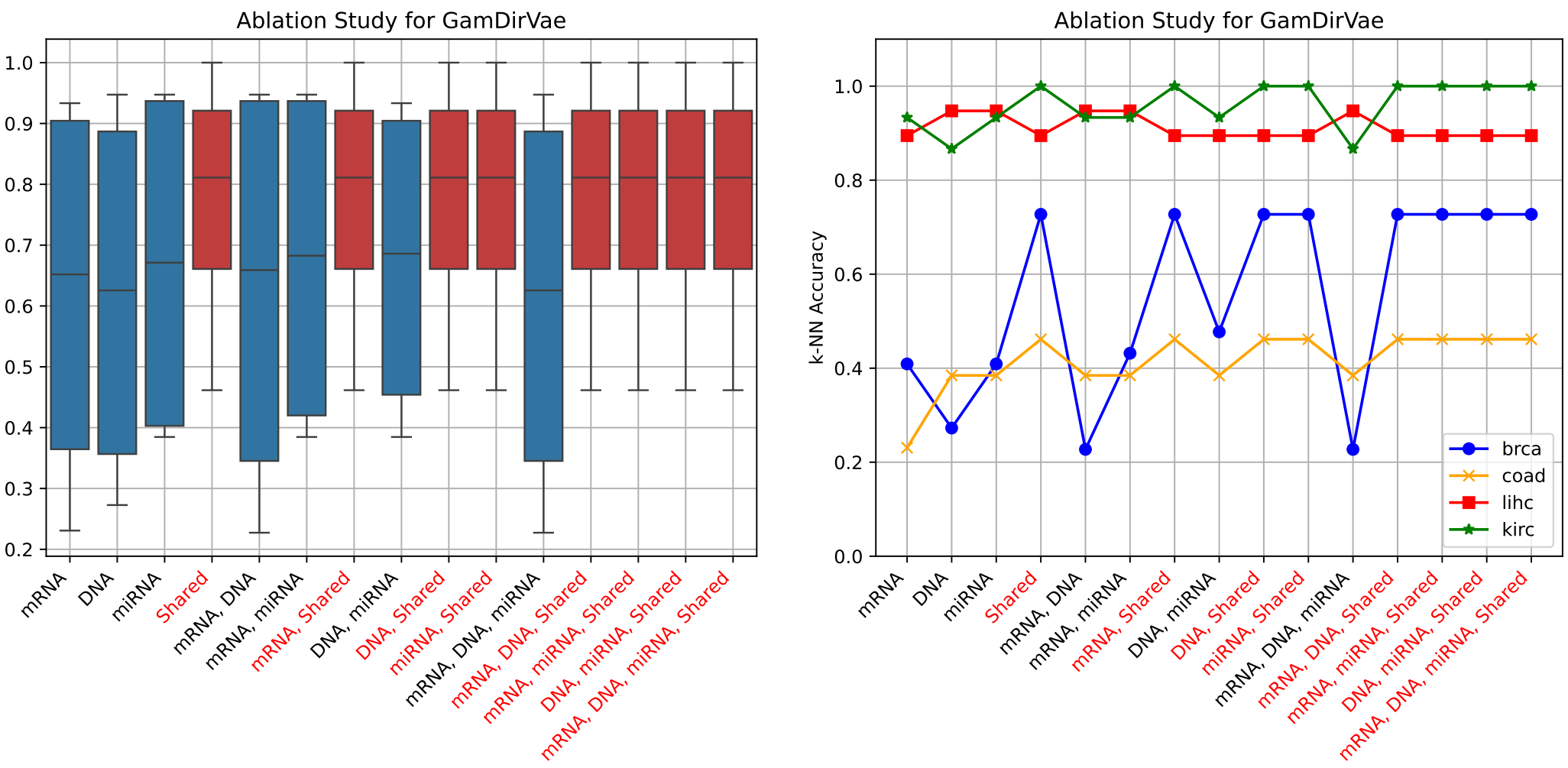

Fig. S17: Ablation results values for accuracy of GamDirVae

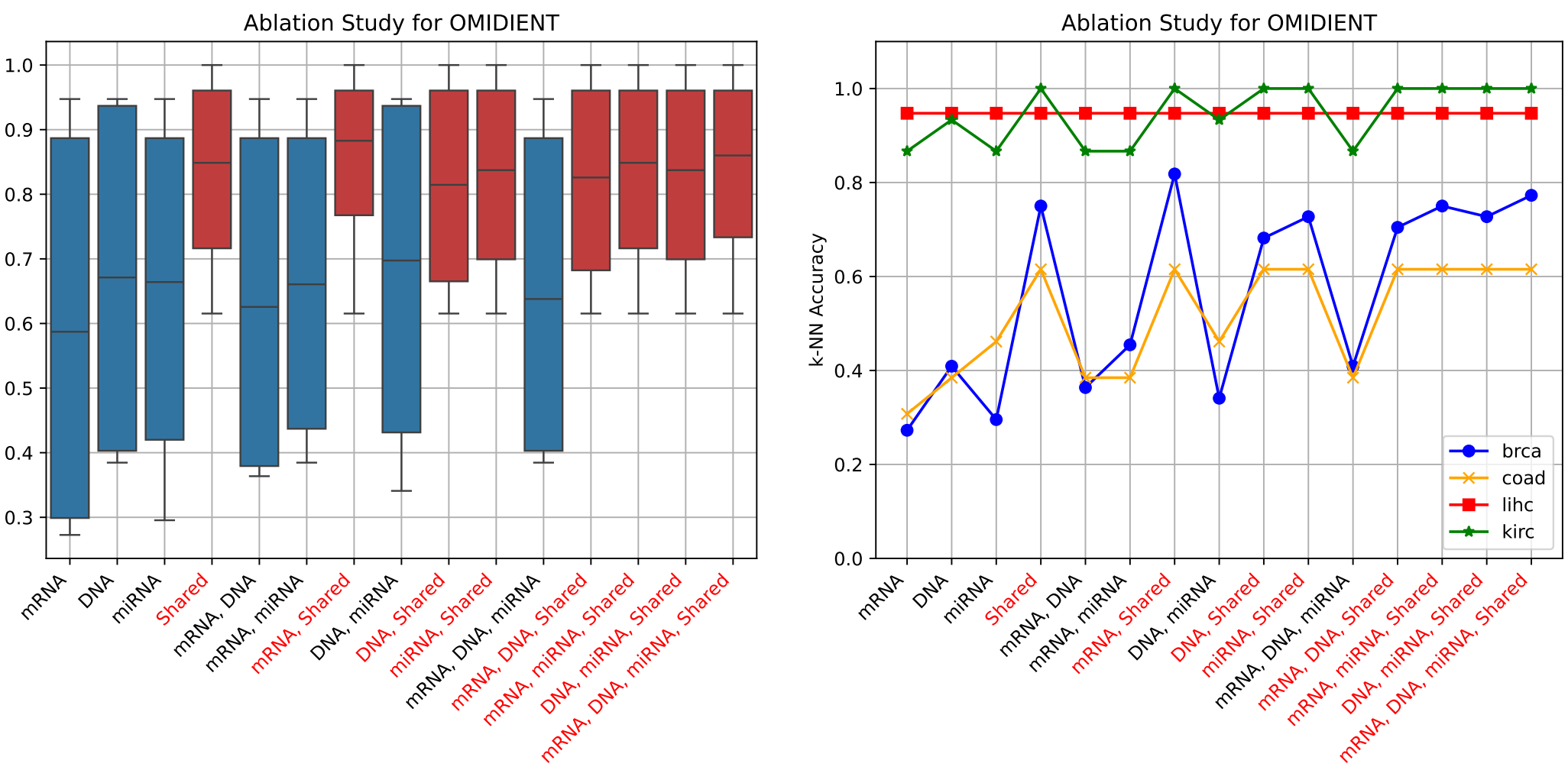

Fig. S18: Shapley values for accuracy of OMIDIENT

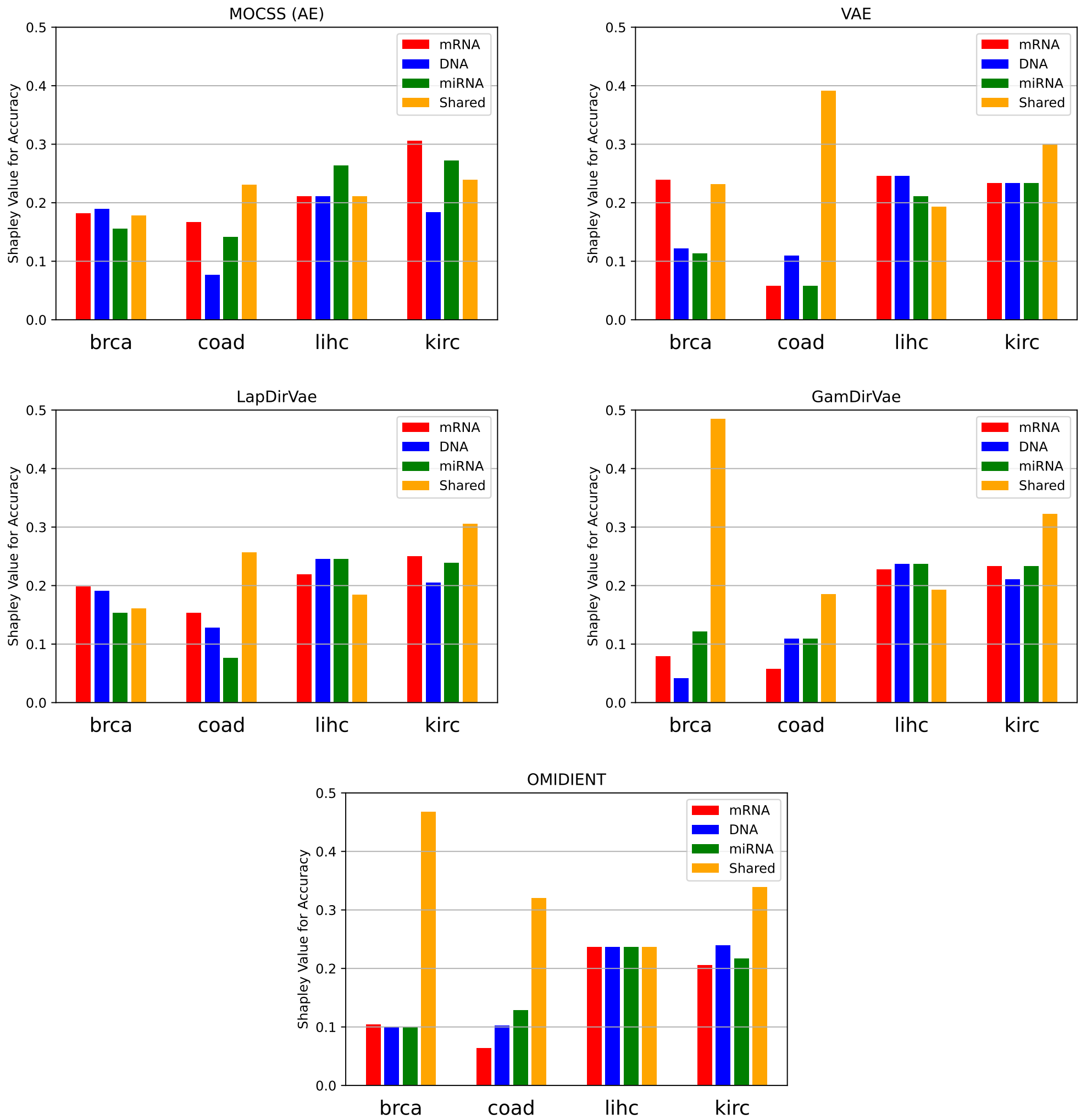

Fig. S19: Shapley values for accuracy of k-NN

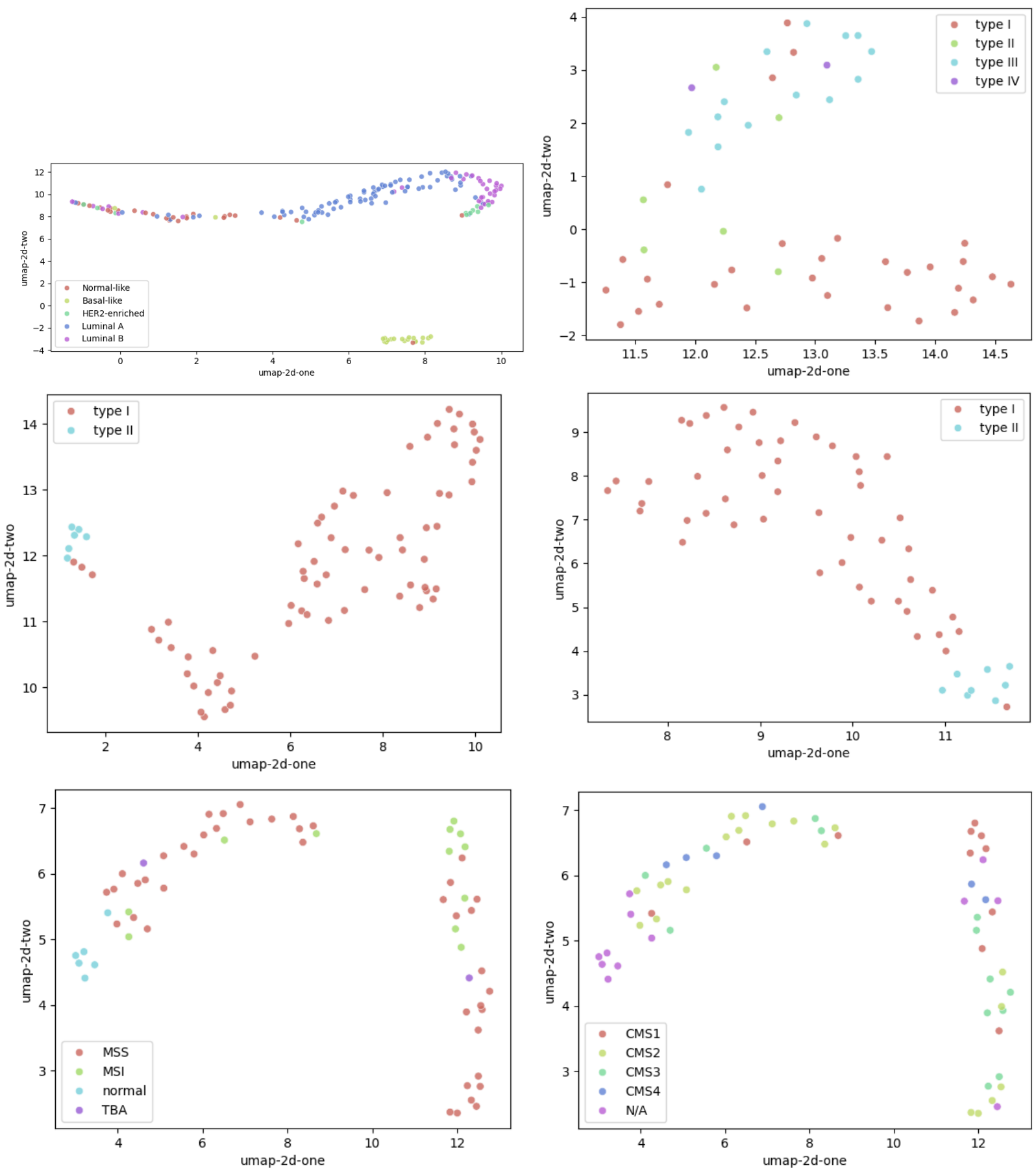

Fig. S20: The class imbalance is visible across the six clustering scenarios, shown from top-left to bottom-right: BRCA, COAD, LIHC, KIRC, MSI labels for CRC, and CMS labels for CRC.

References

[1] Joo, W., Lee, W., Park, S., Moon, I.: Drichlet variational autoencoder. International Conference on Learning Representations (2019)

[2] Bishop, C.M.: Pattern recognition and machine learning. Springer (2006)

[3] Zhang, H., Li, F., Liu, S., Zhang, L., Su, H., Zhu, J., Ni, L., Shum, H.: Dino: Detr with improved denoising anchor boxes for end-to-end object detection. ICLR (2023)

[4] Huang, Z., Shi, J., Gao, Y., Cui, C., Zhang, S., Li, J., Zhou, Y., Cui, Q.: Hmdd v3.0: a database for experimentally supported human microrna-disease associations. Nucleic Acids Res (2019)
