# Supplementary material for: OMIDIENT: Multiomics Integration for Cancer by Dirichlet Auto-Encoder Networks
Source: Commun Biol. 2026 Jul 6;9:1416. doi: 10.1038/s42003-026-10578-1 (PMC13720109; doi:10.1038/s42003-026-10578-1)
Supplement: Supplementary file 2 [file 42003_2026_10578_MOESM8_ESM.pdf]

nature portfolio

Corresponding author(s): Negar Safinianaini

Last updated by author(s): May 19, 2026

Reporting Summary

Nature Portfolio wishes to improve the reproducibility of the work that we publish. This form provides structure for consistency and transparency in reporting. For further information on Nature Portfolio policies, see our Editorial Policies and the Editorial Policy Checklist.

Statistics

For all statistical analyses, confirm that the following items are present in the figure legend, table legend, main text, or Methods section.

n/a	Confirmed	
☐	☒	The exact sample size (*n*) for each experimental group/condition, given as a discrete number and unit of measurement
☐	☒	A statement on whether measurements were taken from distinct samples or whether the same sample was measured repeatedly
☐	☒	The statistical test(s) used AND whether they are one- or two-sided<br>*Only common tests should be described solely by name; describe more complex techniques in the Methods section.*
☒	☐	A description of all covariates tested
☐	☒	A description of any assumptions or corrections, such as tests of normality and adjustment for multiple comparisons
☐	☒	A full description of the statistical parameters including central tendency (e.g. means) or other basic estimates (e.g. regression coefficient) AND variation (e.g. standard deviation) or associated estimates of uncertainty (e.g. confidence intervals)
☒	☐	For null hypothesis testing, the test statistic (e.g. *F*, *t*, *r*) with confidence intervals, effect sizes, degrees of freedom and *P* value noted<br>*Give P values as exact values whenever suitable.*
☐	☒	For Bayesian analysis, information on the choice of priors and Markov chain Monte Carlo settings
☐	☒	For hierarchical and complex designs, identification of the appropriate level for tests and full reporting of outcomes
☒	☐	Estimates of effect sizes (e.g. Cohen's *d*, Pearson's *r*), indicating how they were calculated

Our web collection on statistics for biologists contains articles on many of the points above.

Software and code

Policy information about availability of computer code

Data collection	https://www.cancer.gov/tcga<br>https://api.gdc.cancer.gov<br>https://www.ebi.ac.uk/ega/<br>https://github.com/ChenyuxinXMU/MOCSS<br>https://github.com/GaoLabXDU/MultiOmicsIntegrationStudy/<br>https://acgt.cs.tau.ac.il/multi_omic_benchmark/download.html
Data analysis	For the software we created the below repository with the description of running it in the readme:<br>https://github.com/negar7918/multiomics<br>https://zenodo.org/records/20273710

For manuscripts utilizing custom algorithms or software that are central to the research but not yet described in published literature, software must be made available to editors and reviewers. We strongly encourage code deposition in a community repository (e.g. GitHub). See the Nature Portfolio guidelines for submitting code & software for further information.

Data

Policy information about availability of data

All manuscripts must include a data availability statement. This statement should provide the following information, where applicable:

- Accession codes, unique identifiers, or web links for publicly available datasets
- A description of any restrictions on data availability
- For clinical datasets or third party data, please ensure that the statement adheres to our policy

The BRCA, COAD, LIHC, and KIRC datasets are based upon data generated by The Cancer Genome Atlas (https://www.cancer.gov/tcga). We obtained the preprocessed BRCA and COAD datasets from MOCSS and GaoLab, respectively. For LIHC and KIRC cancers we obtained them from Shamir's Lab . The CRC dataset is deposited in the European Genome-phenome Archive (EGA, https://www.ebi.ac.uk/ega/) for controlled accesses; the study accession number is EGAS00001000854.

Research involving human participants, their data, or biological material

Policy information about studies with human participants or human data. See also policy information about sex, gender (identity/presentation), and sexual orientation and race, ethnicity and racism.

Reporting on sex and gender	sex attribute is used for CRC case to show the interpretability results.
Reporting on race, ethnicity, or other socially relevant groupings	N/A
Population characteristics	genomic information is used for analysis of the results as well as cancer subtyping annotations.
Recruitment	There was no new data and everything was from the patients in the past with their data available publicly.
Ethics oversight	For the public CRC data we had an ethical contract to access the data.

Note that full information on the approval of the study protocol must also be provided in the manuscript.

Field-specific reporting

Please select the one below that is the best fit for your research. If you are not sure, read the appropriate sections before making your selection.

☒ Life sciences ☐ Behavioural & social sciences ☐ Ecological, evolutionary & environmental sciences

For a reference copy of the document with all sections, see nature.com/documents/nr-reporting-summary-flat.pdf

Life sciences study design

All studies must disclose on these points even when the disclosure is negative.

Sample size	Due to the small size of data available, we did not choose any other size than the full size of the data.
Data exclusions	We did not exclude any data from the available data.
Replication	All results are reproducible due to the use of random seed that is hard coded.
Randomization	For training, validation and test we performed a random sampling of the data using a seed.
Blinding	I do not know whether the public data have been under such process or not.

Reporting for specific materials, systems and methods

We require information from authors about some types of materials, experimental systems and methods used in many studies. Here, indicate whether each material, system or method listed is relevant to your study. If you are not sure if a list item applies to your research, read the appropriate section before selecting a response.

Materials & experimental systems

n/a	Involved in the study
☒	☐ Antibodies
☒	☐ Eukaryotic cell lines
☒	☐ Palaeontology and archaeology
☒	☐ Animals and other organisms
☒	☐ Clinical data
☒	☐ Dual use research of concern
☒	☐ Plants

Methods

n/a	Involved in the study
☒	☐ ChIP-seq
☒	☐ Flow cytometry
☒	☐ MRI-based neuroimaging

Plants

Seed stocks	*Report on the source of all seed stocks or other plant material used. If applicable, state the seed stock centre and catalogue number. If plant specimens were collected from the field, describe the collection location, date and sampling procedures.*
Novel plant genotypes	*Describe the methods by which all novel plant genotypes were produced. This includes those generated by transgenic approaches, gene editing, chemical/radiation-based mutagenesis and hybridization. For transgenic lines, describe the transformation method, the number of independent lines analyzed and the generation upon which experiments were performed. For gene-edited lines, describe the editor used, the endogenous sequence targeted for editing, the targeting guide RNA sequence (if applicable) and how the editor was applied.*
Authentication	*Describe any authentication procedures for each seed stock used or novel genotype generated. Describe any experiments used to assess the effect of a mutation and, where applicable, how potential secondary effects (e.g. second site T-DNA insertions, mosiacism, off-target gene editing) were examined.*
