# Supplementary material for: OMIDIENT: Multiomics Integration for Cancer by Dirichlet Auto-Encoder Networks
Source: Commun Biol. 2026 Jul 6;9:1416. doi: 10.1038/s42003-026-10578-1 (PMC13720109; doi:10.1038/s42003-026-10578-1)
Supplement: Supplementary file 3 [file 42003_2026_10578_MOESM2_ESM.docx]

**Description of Additional Supplementary File**

File name: Supplementary data 1
Description: The source data behind the UMAP plots in the paper and supplementary file.

File name: Supplementary data 2
Description: The source data for Fig. 2 in the paper.

File name: Supplementary data 3
Description: The source data for ablation studies of BRCA and COAD cancers.

File name: Supplementary data 4
Description: The source data for ablation studies of KIRC cancer.

File name: Supplementary data 5
Description: The source data for ablation studies of LIHC cancer.
